# Integrated Single‐Cell and Spatial Transcriptomics Reveal Cell‐Type‐Specific Immune Regulatory Networks in Maize Responding to Southern Corn Rust

**DOI:** 10.1002/advs.202512295

**Published:** 2026-03-16

**Authors:** Qiongqiong Wang, Xinyan Sun, Yingchao Sun, Zeqiang Cheng, Zixiang Cheng, Shengbo Han, Ying Feng, Wenbo Yang, Huimin Li, Meichen Zhu, Xiaoling Wu, Jinghua Zhang, Jihua Tang, Honglian Li, Yanyong Cao, Canxing Duan, Yan Shi

**Affiliations:** ^1^ Institute of Cereal Crops Henan Academy of Agricultural Sciences The Shennong Laboratory Zhengzhou China; ^2^ College of Plant Protection State Key Laboratory of High‐Efficiency Production of Wheat‐Maize Double Cropping Henan Agricultural University Zhengzhou China; ^3^ Institute of Crop Sciences Chinese Academy of Agricultural Sciences/State Key Laboratory of Crop Gene Resources and Breeding Beijing China; ^4^ State Key Laboratory of High‐Efficiency Production of Wheat‐Maize Double Cropping Collaborative Innovation Center of Henan Grain Crops College of Agronomy Henan Agricultural University Zhengzhou China; ^5^ State Key Laboratory of High‐Efficiency Production of Wheat‐Maize Double Cropping, College of Life Sciences Henan Agricultural University Zhengzhou China

**Keywords:** *Puccinia polysora*‐maize interaction, immune regulatory networks, snRNA‐seq, stRNA‐seq, cell‐cell communication

## Abstract

Southern corn rust (SCR), caused by *Puccinia polysora* Underw. (*P. polysora*), poses a significant threat to maize production, yet the cell‐type‐specific defense mechanisms remain insufficiently characterized. To address this, we integrated single‐nucleus RNA sequencing (snRNA‐seq) and spatial transcriptomic sequencing (stRNA‐seq) to elucidate the cell‐type‐specific transcriptional dynamics in maize leaves during early infection with *P. polysora*. Analyses at 24 and 48 h post infection (hpi) revealed eight major cell types and highlighted key defense responses, which are primarily initiated in the mesophyll and epidermal cells 24 hpi. Notably, the cell‐type‐specific activation of RLPs/RLKs and jasmonic acid was observed. Functional defense modules were activated in specific cell types at 24 hpi, with pseudotime and cell‐cell communication analyses further uncovering immune‐related cellular dynamics. Importantly, multi‐omics analysis identified core DEGs across critical cell types and time points. Functional validation through virus‐induced gene silencing (VIGS) demonstrated that silencing the *ZmXET1* gene significantly reduced disease severity and pathogen biomass, while silencing the positive regulator *ZmRBG*, increased susceptibility. This study provides a high‐resolution spatiotemporal atlas of maize defense against *P. polysora*, identifying *ZmXET1* as a key susceptibility factor and *ZmRBG* as a resistance component, thereby offering valuable targets for disease resistance breeding.

## Graphical abstract

By integrating single‐nuclei and spatial transcriptomics, this study presents a stereoscopic landscape of maize leaf to *Puccinia polysora* infection. Epidermal and mesophyll cells initiate primary defenses via RLPs/RLKs and jasmonic acid signaling. Cell‐cell communication analyses further reveal the underlying the dynamics of the underlying immune network.

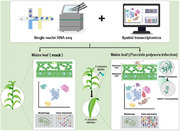

## 1 Introduction

Maize (*Zea mays* L.) is a globally significant crop, serving as a vital source of food, feed, and industrial products [1]. However, *Puccinia polysora* Underw. (*P. polysora*), the causative agent of southern corn rust (SCR), represents a major threat to maize production [2, 3]. This disease leads to substantial yield losses, typically ranging from 30% to 50%, and in severe cases, can cause over 50% reduction in yield, thereby negatively impacting both the quality and quantity of maize harvests and posing a serious challenge to global food security [4]. Thriving in humid environments, the pathogen infects maize leaves and rapidly disseminates throughout the plant, impairing photosynthesis and hindering growth [4, 5, 6, 7]. As the economic burden of SCR increases, research focusing on the dynamic allocation of resources for maize in response to this infection has become essential [6, 8]. and understanding how maize directs its defense resources at both the whole‐plant and cellular levels is critical for developing effective SCR management strategies.

SCR pathogenicity in maize involves complex interactions between the pathogen and the immune system [4], with the genomic characteristics of *P. polysora* revealing several effector proteins that manipulate the host physiology to facilitate infection [7, 9]. These effectors modulate the host immune responses by interfering with signaling pathways and suppressing defense mechanisms via a temporally and spatially organized infection process [3, 4, 6, 10, 11, 12, 13]. *P. polysora* initially infects the leaf surface, and then penetrates the plant, progressing through various tissue layers [4, 14]. Emerging evidence suggests the host response is dynamic, with studies showing distinct tissues and cell types exhibiting differential immune responses at different stages of infection [11, 15, 16]. *ZmREM1.3* positively regulates maize defenses against *P. polysora*, likely through the salicylic acid (SA)/jasmonic acid (JA)‐mediated signaling pathways [11]. Moreover, the maize NLR gene *RppK*, which confers resistance to SCR, has been successfully cloned along with the corresponding *Avr* gene, *AvrRppK*, from *P. polysora*. The *RppK* gene provides broad‐spectrum resistance against SCR by recognizing the core effector protein *AvrRppK* [10]. Other defense proteins, such as *RppC* and *RppM*, have also been identified and studied [2, 12, 13]. Despite these advances, research into the defense mechanisms that maize uses against *P. polysora* and the intricate regulatory networks remains limited, hindering progress in the development of effective disease management strategies.

Recent advancements in transcriptomic technologies have transformed the study of the growth, development, and pathogen interactions of plants by providing unprecedented resolution [17, 18, 19, 20, 21], with two emerging methodologies, single‐cell transcriptome sequencing (scRNA‐seq) and spatial transcriptome sequencing (stRNA‐seq), offering complementary advantages in this regard [21, 22, 23]. The scRNA‐seq enables gene expression analysis at the individual cell level, revealing the cellular heterogeneity of plant responses to pathogen attacks [16, 19, 24], and researchers have employed both single‐cell and spatial transcriptomics to elucidate the multifaceted response in the leaf cells of rice to *Magnaporthe oryzae* infection [24]. This high‐resolution approach identifies the specific cell types that are involved in the defense response and captures any dynamic gene expression changes during infection [25]. In contrast, stRNA‐seq provides a map of the gene expression within plant tissues, facilitating the examination of tissue‐specific and time‐dependent variations [26, 27, 28, 29]. The integration of both techniques thus offers a comprehensive perspective on the molecular mechanisms that drive the plant immune response, uncovering how different tissues and cell types collaborate to fend off pathogens.

Although both single‐cell and spatial transcriptome sequencing have been extensively utilized to investigate plant growth and development [22, 30, 31], the application of these techniques to plant‐pathogen interactions remains limited. The integration of these technologies holds great potential for enhancing our understanding of the interactions between maize and *P. polysora*; thus, this study leveraged single‐nucleus RNA sequencing (snRNA‐seq) and stRNA‐seq to investigate the dynamic interplay between maize and *P. polysora* during infection. By combining these advanced techniques, the early‐stage defense dynamics of maize against *P. polysora* were characterized and the key genes and pathways involved in the plant immune responses were identified, including pattern‐triggered immunity (PTI), effector‐triggered immunity (ETI), and the JA and SA signaling pathways. Furthermore, critical defense networks and hub genes were pinpointed, elucidating the roles of *ZmXET1* and *ZmRBG* in maize defense. This study thus offers a comprehensive overview of the immune processes used by maize against *P. polysora*, providing valuable insights into the molecular mechanisms underlying maize defense and identifying key genes that could inform future resistance breeding strategies.

## 2 Results

### 2.1 The Infection Establishment of *P. polysora* in Maize Seedling Leaves

**FIGURE 1:**
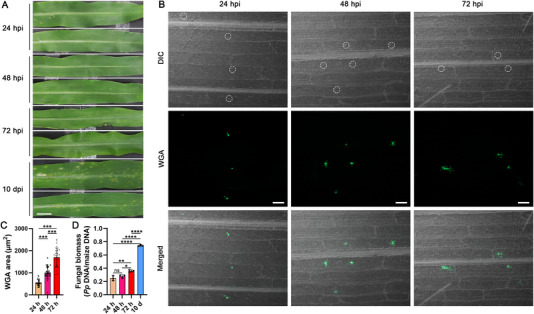
Establishment of *P. polysora* infection in maize leaves. (A) Development of symptoms on maize leaves inoculated with *P. polysora* 24 hpi, 48 hpi, 72 hpi, and 10 dpi. Scale bar = 1 cm. (B) Infection process of *P. polysora* monitored by WGA‐AF488 staining. Fungal hyphae WGA‐AF488 stained with substomatal vesicles with marked by green fluorescence. The dashed circle indicates the stoma where urediniospores were germinated and hyphae penetrated. Scale bars = 100 µm. DIC, differential interference contrast. (C) The WGA signals were evaluated by measuring the area density of the green fluorescence. *** *p* < 0.001, significance determined by ordinary one‐way ANOVA with Tukey's HSD post hoc test for pairwise comparisons (*n* = 30 leaves) (D) Relative fungal biomass assessment in *P. polysora*‐inoculated maize leaves. Significant differences in fungal biomass among sites were assessed by one‐way ANOVA with Tukey's HSD post hoc test. Significance levels are denoted as * *p* < 0.05, ** *p* < 0.01, **** *p* < 0.0001; ns, not significant. Error bars represent mean ± SD.

The aim of this study was to identify the critical early time point at which *P. polysora* infection onset and to track its early progression of the disease in maize leaves. The resistant inbred line Qi319 was inoculated with *P. polysora* spores and infection progression evaluated at multiple time points using both phenotypic and histological analyses. No visible symptoms were observed on Qi319 leaves at 24 hpi, 48 hpi, or 72 hpi (Figure 1A). However, fluorescent microscopy using wheat‐germ agglutinin (WGA) staining demonstrated successful fungal infection by 24 hpi, as evidenced by the formation of substomatal vesicles, which is a hallmark structure of rust fungal invasion and signifies the successful entry of the pathogen via either direct penetration of the leaf epidermis or through the stomata, and into the intercellular spaces of the plant tissue (Figure 1B). This is accompanied by the development of primary hyphae and the formation of haustorial mother cells. (Figure 1B). Increased branching and colonization of the fungal hyphae was observed within the intercellular spaces along with the formation of haustoria at 48 hpi, facilitating nutrient uptake from the host cells, and by 72 hpi, fungal growth was further advanced, with the cellular environment undergoing significant changes, such as the distortion of stomatal structures, although still without visible symptoms (Figure 1B). Scattered sporulation in the form of mature lesions became evident on the leaf surface 10 days post‐inoculation (dpi) (Figure 1A), and quantification via WGA staining and the fungal biomass further confirmed a progressive increase in the fungal load from 48 hpi to 72 hpi, as supported by statistical analysis (Figure 1C,D).

The selection of 24 hpi and 48 hpi as key early time points was based on prior histological evidence [15], which delineates 48 hpi as a critical threshold for early biotrophic invasion before widespread host defense responses such as phenolic deposition and organelle disruption begin, which become dominant at 72 hpi. Thus, the transition from initial fungal establishment to early host engagement can be captured at 48 hpi, rendering this point particularly informative for studying pre‐defense molecular responses and establishing 24 hpi and 48 hpi as crucial early time points for investigating early infection with *P. polysora* in maize leaves.

### 2.2 Establishment of Cell Types in *P. polysora*‐infected Maize Leaves

**FIGURE 2:**
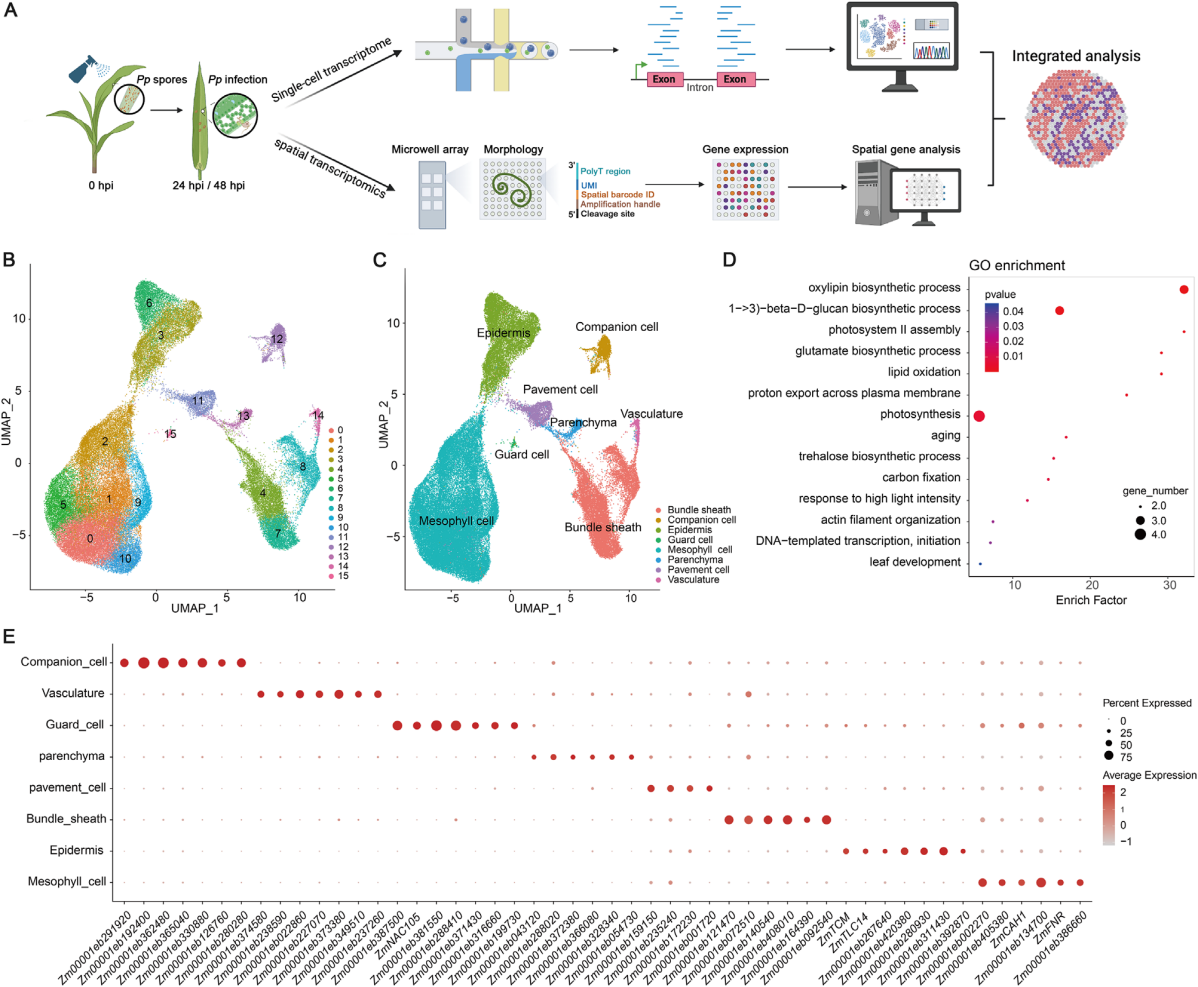
Transcriptome atlas of maize leaf cells infected with *P. polysora*. (A) Workflow analysis for snRNA and stRNA sequencing of *P. polysora* infected maize leaves 24 hpi and 48 hpi. (B) Expression analysis of cells sequenced using snRNA‐seq, followed by dimensionality reduction and clustering. The X‐axis and Y‐axis represent data mapped following dimensionality reduction. (C) Identification of cell types in different clusters of snRNA‐seq using a relevant marker gene database. (D) GO enrichment analysis of the identified mesophyll cell genes, with the X‐axis representing Enrichment Factors, and the Y‐axis indicating the GO term. (E) Bubble plot of marker genes identified in different cell types. The X‐axis lists the marker genes, the Y‐axis represents leaf cell types, the points size indicates the frequency of gene expression across all cells in that tissue, and color reflects the expression level of the gene in the investigated tissue.

The establishment of a transcriptional landscape of *P. polysora* infection on maize leaves at various time points was also sought. The snRNA‐seq and stRNA‐seq were thus performed on samples collected 24 hpi and 48 hpi (Figure 2A). A total of 12 single‐cell samples were collected, at two time points for *P. polysora* treated leaves (PP24h and PP48h) and the mock treatment (MK24h and MK48h). Nuclei were isolated and filtered to construct snRNA‐seq libraries, which were then sequenced using 10× Genomics technology. This yielded a total of 131 601 high‐quality single cells, with an average of 30 424 genes detected per sample and a median of 977 genes detected per cell (Figure S1A; Table S1). The snRNA‐seq data showed strong correlations across the three biological replicates for each sample, validating the robustness of the results (Figure S1B). After data normalization and linear dimension reduction, various resolution parameters were tested to determine the optimal settings for cell clustering, which were then applied to ensure the accurate clustering and visualization of cell types using Uniform Manifold Approximation and Projection (UMAP) [32], resulting in the identification of 16 major cell clusters (Figure 2B; Figure S2 and Table S2).

To identify the cell types associated with the major cell clusters in maize leaves, marker genes for the 16 major cell clusters were first elucidated, with a total of 3922 marker genes distributed across different clusters were observed (Table S3). Published marker genes related to maize leaves were also incorporated [18], together with data from public databases such as PlantCellMarker [33], PlantscRNAdb [34], and PsctH [35] to classify the cell types of the 16 major clusters. The results revealed that the 16 clusters could be grouped into 8 distinct cell types (Figure 2C). Specifically, the marker genes for Clusters 0, 1, 2, 5, 9, and 10 were identified as mesophyll cells, while Clusters 3 and 6 were classified as epidermal cells, Clusters 4, 7, and 8 were associated with bundle sheath cells, and Clusters 11, 12, 13, 14, and 15 were annotated as pavement cells, companion cells, parenchyma, vasculature, and guard cells, respectively. Both pavement cells and guard cells are specialized epidermal cell types [36] (Figure 2C). To further explore the functional roles of the different cell types, Gene Ontology (GO) enrichment analysis was performed on the mesophyll cells, the largest cell type group, with the results revealing that the differentially expressed genes (DEGs) in mesophyll cells were primarily involved in oxylipin biosynthesis, (1,3)‐beta‐D‐glucan biosynthesis, and photosynthesis (Figure 2D; Tables S4 and S5). GO enrichment analysis across all cell types highlighted functional differences, validating the functional annotation of the cell populations (Figure S3). Several marker genes associated with the specific cell types were also identified (Figure 2E). For example, *Zm00001eb158810* (*ZmCAH1*, *Carbonic anhydrase1*) and *Zm00001eb362640* (*ZmFNR, Ferredoxin‐NADP reductase*) were identified as markers for mesophyll cells, while *Zm00001eb232100* (*ZmTCM, Terpene cyclase/mutase*) and *Zm00001eb333330* (*ZmTLC14, TRAM LAG1 and CLN8 lipid‐sensing gene*) showed high expression in epidermal cells, and *Zm00001eb387500* and *Zm00001eb173960* (*ZmNAC105, NAC‐transcription factor 105*) highly expressed in guard cells (Figure S4A). To further validate these candidate marker genes in vivo, RNA in situ hybridization was conducted, confirming the reliability of the identified cell type‐specific markers for distinguishing maize leaf cell types (Figure S4B). An expression profile of maize leaves was thus constructed using single‐cell data, successfully identifying subcellular types and their associated marker genes.

### 2.3 Visualization of *P. polysora* Infection in Maize Leaves via the Spatial Transcriptomic Atlas

**FIGURE 3:**
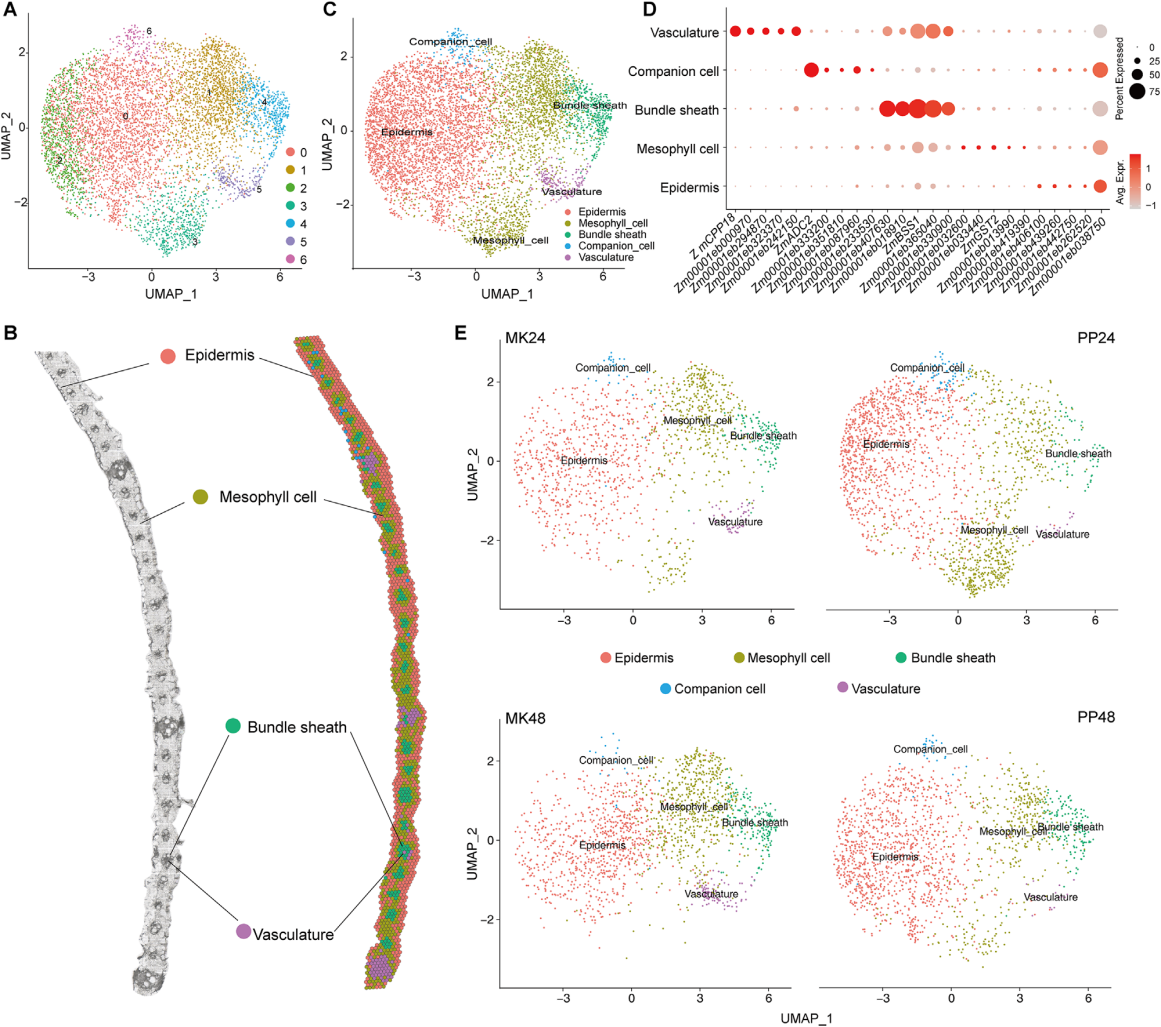
Spatial transcriptomic atlas of maize leaves infected with *P. polysora*. (A) Expression profiling, dimensionality reduction, and clustering of cells sequenced by stRNA‐seq. The X‐axis and Y‐axis represent the coordinates following dimensionality reduction. (B) Spatial mapping and distribution of distinct cell types within a leaf sample. (C) Assignment of transcriptomic clusters to specific cell types. The X‐axis and Y‐axis are identical to those in panel A. (D) Bubble plot depicting marker genes identified across different cell types via stRNA‐seq. The X‐axis lists marker genes, the Y‐axis corresponds to leaf cell types, bubble size indicates the fraction of expressing cells within the tissue, and color represents the average expression level. (E) Distribution of stRNA‐seq‐derived cell types across experimental conditions.

Spatial transcriptomics technology allows for the high‐resolution visualization of tissue specificity and spatial expression variations [22]. This study thus aimed to examine the spatial expression dynamics of *P. polysora* infection in maize leaves using stRNA‐seq. Maize leaves infected with *P. polysora* were frozen in isopentane and liquid nitrogen baths, followed by embedding in OCT and sectioning with a freezing microtome. The optimal permeabilization time was determined, and stRNA‐seq was subsequently performed (Figure 2A). After data filtering, 21 388 spots were obtained, with the median gene count per spot ranging from 638 to 2 274. The average number of detected genes was 24 292 (Table S6). Linear regression and UMAP visualization were applied to the stRNA‐seq data, resulting in the categorization of the detected spots into 7 clusters (Figure 3A; Figure S5). Unlike snRNA‐seq, the analysis leverages the spatial coordinates of each cluster, allowing them to be mapped to distinct anatomical locations within the tissue—a key advantage for differentiating region‐specific gene expression patterns. Consequently, various subcellular structures within the cross‐section of the maize leaf were clearly resolved and visualized, with their corresponding gene expression profiles captured by associated probes (Figure 3B). By integrating these spatial coordinates with established marker genes for validation, we annotated the seven clusters into five major leaf cell types: clusters 0 and 2 were identified as epidermis, clusters 1 and 3 as mesophyll, cluster 4 as bundle sheath, cluster 5 as vasculature, and cluster 6 as companion cells (Figure 3C).

To further characterize the cell types associated with different clusters, genes highly expressed in each cluster were defined as marker genes (Figure 3D; Table S7). For instance, *Zm00001eb169010* (*ZmCPP18*, *cysteine protease 18*) was highly expressed in vasculature, *Zm00001eb185400* (*ZmADC2*, *arginine decarboxylase 2*) was predominantly expressed in companion cells, *Zm00001eb362480* (*ZmBSS1, bundle sheath strands specific 1)* in bundle sheath, and *Zm00001eb418060* (*ZmGST2*, *glutathione S‐transferase 2*) in mesophyll cells (Figure S6). Analysis of gene expression under different treatment conditions revealed that the expression of cell type‐specific genes in both epidermal and mesophyll cells increased significantly at 24 and 48 h after *P. polysora* treatment (Figure 3E). These results suggest that epidermal and mesophyll cells play important roles in the maize leaf's response to *P. polysora* infection.

### 2.4 Cell Type‐specific Differential Expression of Immune‐related Genes

**FIGURE 4:**
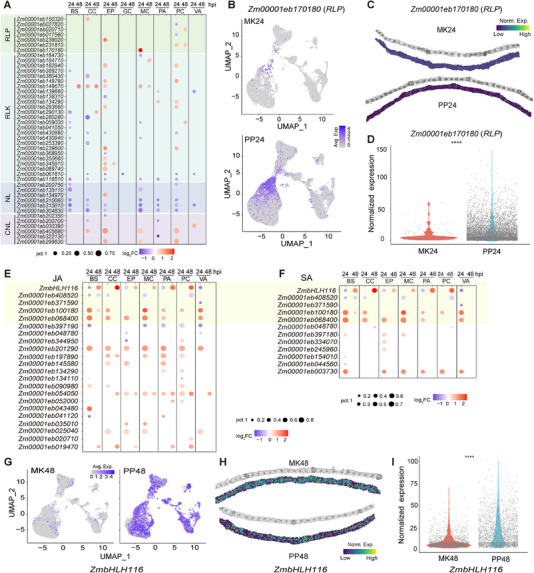
Cell type‐specific differential expression of immune‐related genes. (A) Identification of the expression of PTI and ETI‐related genes in snRNA‐seq across eight leaf tissues (BS: bundle sheath cells, CC: companion cells, EP: epidermal cells, GC: guard cells, MC: mesophyll cells, PA: pavement cells, VA: vasculature) at two time points (24 hpi and 48 hpi). Point size represents the normalized expression ratio, while color indicates the log_2_ fold change (log_2_FC) in the DEGs as compared to the control at each time point. (B) Expression of the *Zm00001eb170180* gene 24 hpi in different clustering locations as obtained via snRNA‐seq from treatment and control groups. (C) Expression levels and locations of the *Zm00001eb170180* gene in stRNA‐seq at 24 h in the treatment and control groups. (D) Violin plot showing the expression level of *Zm00001eb170180* in mesophyll cells from stRNA‐seq data; each point represents an individual cell. Statistical analysis was performed using Student's *t*‐test: ****p < 0.0001, *n* = 175 (MK24) and 7190 (PP24) cells. (E) Identification of expression of JA pathway‐associated genes in maize across different tissues obtained via snRNA‐seq. (F) Identification of expression of SA pathway‐associated genes in maize across different tissues obtained via snRNA‐seq. (G) Expression of *Zm00001eb415520* in different clustering locations obtained via snRNA‐seq at 24 h in the treatment and control groups. (H) Expression levels and locations of the *Zm00001eb415520* gene obtained via stRNA‐seq at 24 h in the treatment and control groups. (I) Violin plot showing the expression level of *Zm00001eb415520* in mesophyll cells from stRNA‐seq data; each point represents an individual cell. Statistical analysis was performed using Student's *t*‐test: ****p < 0.0001, *n* = 1793 (MK24) and 2448 (PP24) cells, respectively.

The PTI and ETI pathways are central to plant immune responses [37, 38]. Thus, the immune‐related genes in maize were systematically identified (see Experimental Section). Two gene families were associated with Pattern Recognition Receptors (PRRs): Receptor‐Like Proteins (RLPs) and Receptor‐Like Kinases (RLKs). Their expression exhibited cell type‐specific patterns in the maize leaves (Figure 4A). At 24 h post‐inoculation with *P. polysora*, distinct expression patterns of RLPs and RLKs were observed, primarily within mesophyll and epidermal cells (Figure 4A). In particular, the expression of the RLP gene *Zm00001eb170180* was most markedly altered in mesophyll cells at this time point (Figure 4B). Spatial transcriptomics confirmed this finding, revealing a concurrent increase in both expression level and the number of positive mesophyll cells (Figure 4C,D). In contrast, RLK genes were predominantly upregulated in the epidermal and pavement cells 24 hpi (Figure 4A). Resistance genes (R‐genes) are primarily categorized into NL (NBS‐LRR) genes and CNL (CC‐NBS‐LRR) genes [39], and the results revealed that most NL genes were downregulated following *P. polysora* infection (Figure 4A), and that while CNL genes exhibited expression patterns spatially similar to NL genes, distinct clusters of CNL genes showed upregulated expression under the same conditions (Figure 4A).

Plant hormones, particularly JA and SA, are key regulators in pathogen defense [24, 40, 41]. Therefore, genes associated with the JA and SA signaling pathways in maize were also characterized and analyzed, with results indicating that genes from both hormonal pathways did not display prominent cell type‐specific responses, and that most showed consistent expression patterns across all cell types (Figure 4E, F). Interestingly, similar to the immune‐responsive genes, both hormonal pathways were predominantly activated 24 hpi, with a subset of genes exhibiting delayed regulation 48 hpi (Figure 4E,F). Notably, the shared pathway gene *Zm00001eb415520* (*ZmbHLH116*, which encodes for the transcription factor *bHLH116*) was activated 48 hpi (Figure 4G). Spatial expression profiling further confirmed this regulatory pattern (Figure 4H,I). Collectively, these findings highlight the complex regulatory dynamics of the JA and SA pathways during *P. polysora* infection in maize leaves.

### 2.5 Co‐expression Network Analysis Reveals Core Functional Modules Linked to *P. polysora* Infection in Maize Leaves

**FIGURE 5:**
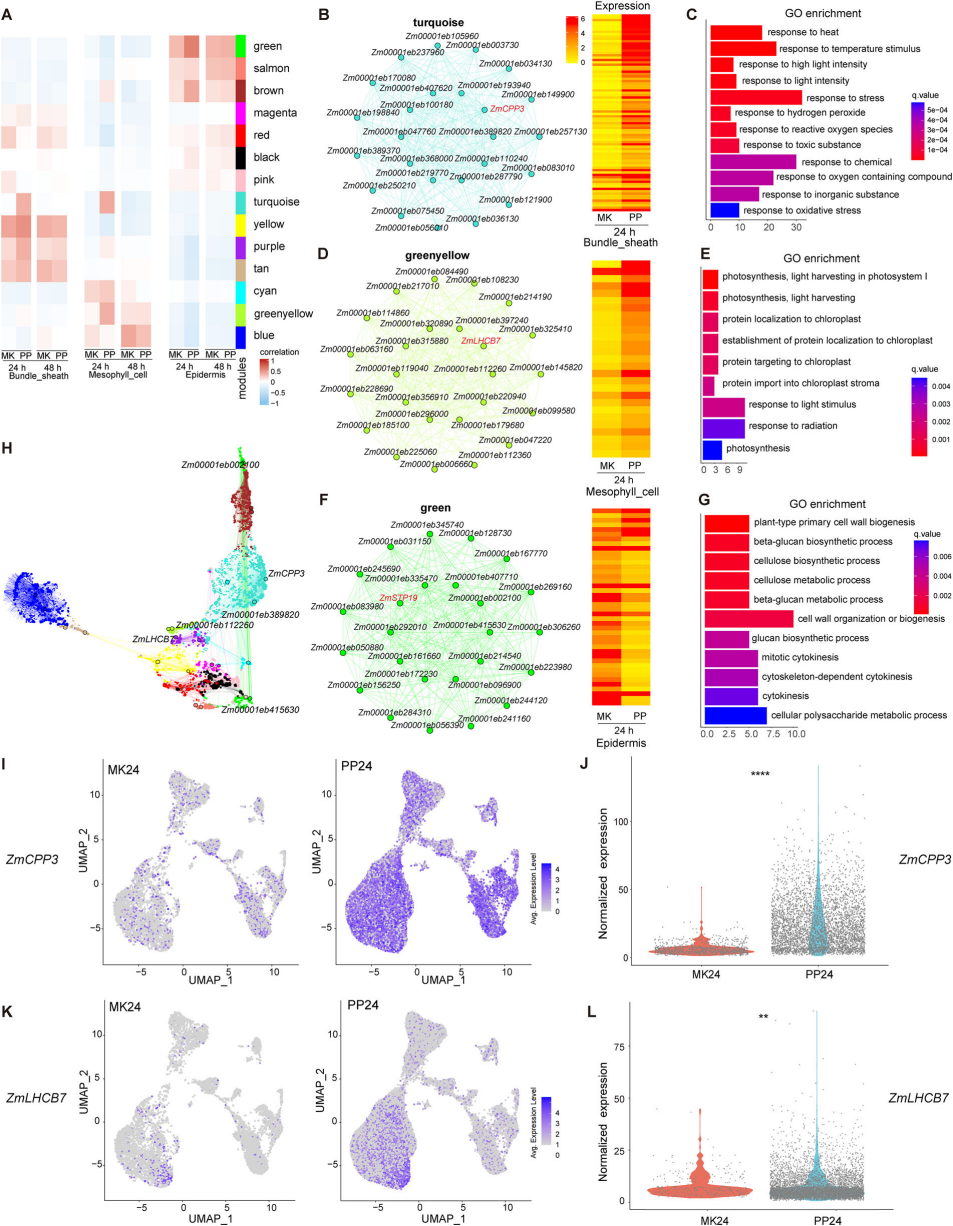
Co‐expression module reveals key functional modules associated with *P. polysora* infection in maize leaves. (A) Correlation analysis of different modules and cell types observed via WGCNA analysis under different treatments. Red indicates a positive correlation, while blue indicates a negative correlation. (B) Left layout: gene network of the turquoise module, right layout: heatmap of relative expression of network nodes genes in treatment and control groups 24 hpi. (C) GO enrichment analysis of relevant genes in the turquoise module. (D) Left layout: gene network of the green‐yellow module, right layout: heatmap of relative expression of network nodes genes in treatment and control groups 24 hpi. (E) GO enrichment analysis of relevant genes in the green‐yellow module. (F) Left layout: gene network of the green module, right layout: heatmap of relative expression of network nodes genes in treatment and control groups 24 hpi. G) GO enrichment analysis of relevant genes in the green module. (H) Network topology that merges multiple modules, with gene IDs indicating hub genes for the relevant modules. (I) Expression of the *ZmCPP3* gene in different clustering locations obtained via snRNA‐seq at 24 h in treatment and control groups. (J) Violin plot showing the expression level of *ZmCPP3* in bundle sheath cells from stRNA‐seq data; each point represents an individual cell. Statistical analysis was performed using Student's *t*‐test: ****p < 0.0001, *n* = 896 (MK24) and 4393 (PP24) cells. (K) Expression of the *ZmLHCB7* gene in different clustering locations obtained via snRNA‐seq at 24 h in treatment and control groups. (L) Violin plot showing the expression level of *ZmLHCB7* in mesophyll cells from stRNA‐seq data; each point represents an individual cell. Statistical analysis was performed using Student's *t*‐test: ****p < 0.0001, *n* = 357 (MK24) and 6292 (PP24) cells.

To investigate the co‐expression networks during *P. polysora* infection in maize leaves, weighted gene co‐expression network analysis (WGCNA) was conducted on cell type‐specific DEGs, with 14 distinct modules identified (Figure S7A). Module‐trait association analysis revealed significant correlations between the specific modules and particular cell type clusters (Figure S7B). Notably, the turquoise, yellow, purple, and tan modules were strongly associated with bundle sheath cells, while turquoise and greenyellow modules correlated with mesophyll cells, and the green module with epidermal cells (Figure 5A). Notably, the connectivity of the turquoise (bundle sheath), greenyellow (mesophyll), and green (epidermal) modules increased significantly in their respective cell types 24 hpi (Figure 5A). Subsequent functional characterization of the modules revealed distinct patterns. Genes within the turquoise module were markedly upregulated 24 hpi (Figure 5B), and GO enrichment analysis showed that these genes are primarily involved in stress responses, such as the oxidative stress response, cellular response to reactive oxygen species (ROS), and responses to oxygen‐containing compounds (Figure 5C). Similarly, the greenyellow module exhibited temporal activation 24 hpi (Figure 5D), with GO enrichment related to photosynthetic processes, such as the photosystem II assembly, chloroplast localization, and light‐harvesting complex organization (Figure 5E). In contrast, genes in the green module showed significant downregulation 24 hpi (Figure 5F), with enrichment in cell wall biogenesis and carbohydrate metabolic processes (Figure 5G). Network topology analysis identified central regulators (hub genes) with high intramodular connectivity (Figure 5H). The hub gene *Zm00001eb432170* (*ZmCPP3*, *cysteine protease 3*) in the turquoise module was transiently upregulated in bundle sheath cells at 24 hpi, while its expression was suppressed by 48 hpi (Figure 5I,J; Figure S8A), while the hub gene *Zm00001eb296090* (*ZmLHCB7*, *light‐harvesting complex mesophyll 7*) in the greenyellow module showed significant upregulation in mesophyll cells 24 hpi and 48 hpi (Figure 5K,L; Figure S8B). Additionally, the hub gene *Zm00001eb312640* (*ZmSTP19*, *sugar transport protein 19*) in the green module was significantly downregulated in epidermal cells 24 hpi, with no further change observed 48 hpi (Figure S8C). In summary, the multi‐module analysis defines a two‐phase defense reprogramming process: 1) the activation of stress‐responsive (turquoise) and photoprotective (greenyellow) pathways in bundle sheath/mesophyll cells at 24 hpi, and 2) the concurrent suppression of epidermal cell wall biosynthesis (green module). These results indicate that maize leaves undergo a spatially coordinated defense reprogramming during early infection, as characterized by the activation of stress and photoprotective pathways in the inner cells (bundle sheath/mesophyll) alongside a transient suppression of the structural defenses in epidermal cells.

### 2.6 Differentiation Trajectories and Annotation Reveal the Importance of Defense Cells

**FIGURE 6:**
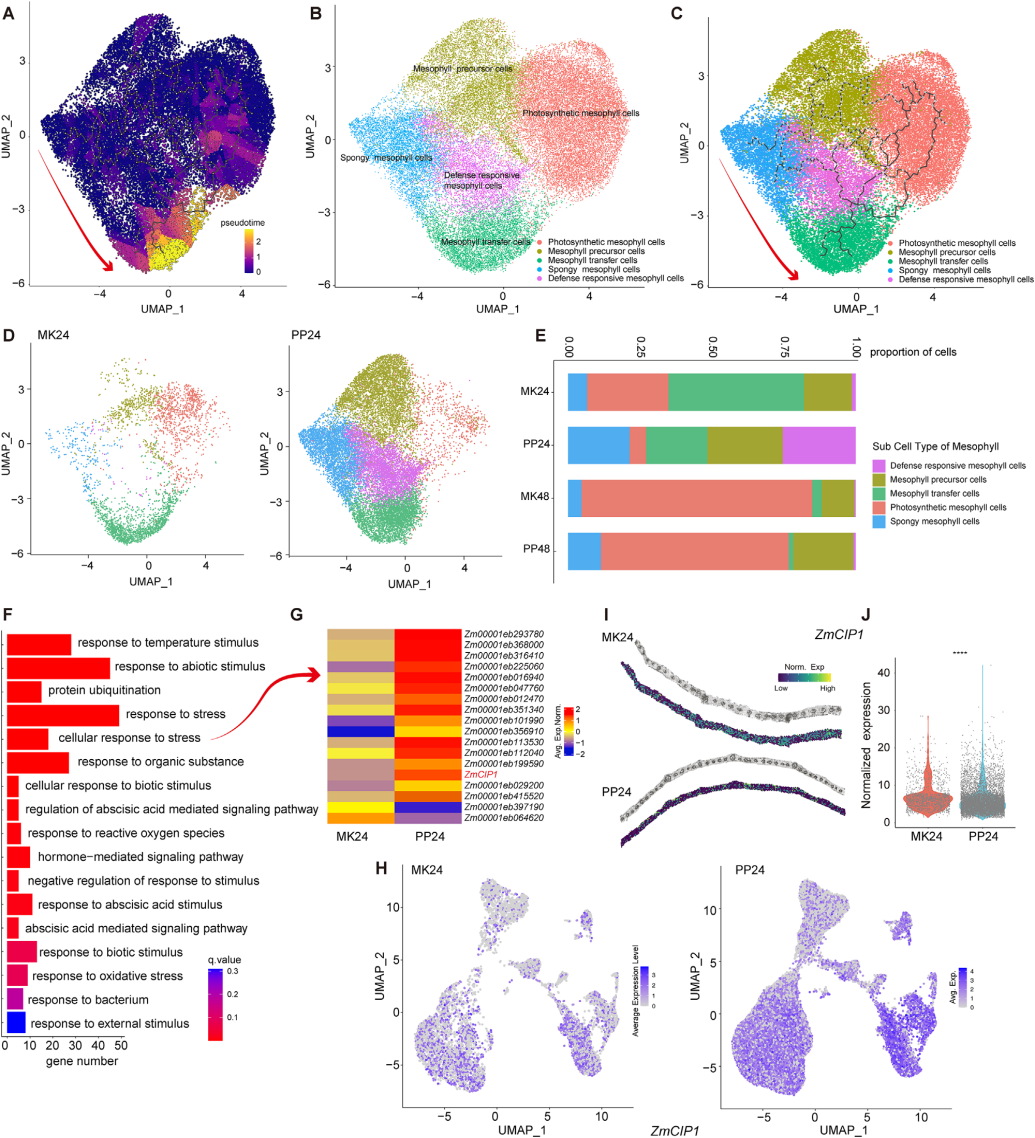
Differentiation trajectories of mesophyll cells in response to *P. polysora*. (A) Trajectory curves of mesophyll cells in maize leaves, where each point represents an individual cell colored by its inferred state. (B) Functional annotation and classification of mesophyll cells, with colors representing distinct putative functional subtypes. (C) Differentiation trajectories of mesophyll cells across putative functional subtypes. (D) Divergence in differentiation trajectories of mesophyll cells across experimental conditions at 24 hpi. (E) C Compositional analysis of functional subtypes in mesophyll cells across samples, shown as a stacked bar plot (X‐axis: proportional distribution of five functional subtypes; Y‐axis: different sample groups). (F) GO functional enrichment analysis of key DEGs from defense‐responsive mesophyll cell subtypes at 24 hpi. (G) Heatmap of key DEGs in stress‐responsive subtypes at 24 hpi. (H) Expression of the *ZmCIP1* gene in different clustering locations obtained via snRNA‐seq at 24 hpi in treatment and control groups. (I) Expression of the *ZmCI1P* gene in different locations obtained via stRNA‐seq at 24 hpi in treatment and control groups. (J) Violin plot showing the expression level of *ZmCIP1* in mesophyll cells from stRNA‐seq data; each point represents an individual cell. Statistical analysis was performed using Student's *t*‐test: ****p < 0.0001, *n* = 624 (MK24) and 9412 (PP24) cells, respectively.

SnRNA‐seq technology facilitates the exploration of the developmental states and differentiation trajectories of plant cells. The above and previous studies have highlighted the critical roles that mesophyll cells play during *P. polysora* infection in maize leaves [15]. Thus, to deepen our understanding of the immune response in maize, the gene expression trajectories of mesophyll cells were reconstructed using pseudotime analysis (Figure 6A). Concurrently, we annotated the mesophyll cell population based on distinct functional subtypes. This analysis enabled the classification of mesophyll cells into several groups: photosynthetic mesophyll cells, mesophyll precursor cells, mesophyll transfer cells, spongy mesophyll cells, and defense‐responsive mesophyll cells (Figure 6B). Integrated pseudotime analysis revealed a cellular trajectory progressing from spongy mesophyll cells through defense‐responsive mesophyll cells and culminating in mesophyll transfer cells (Figure 6C). Furthermore, differentiation trajectories were separately constructed for *P. polysora*‐infected samples and mock‐treated controls. The results showed a significant divergence between the trajectories at 24 hpi, whereas the difference was minimal at 48 hpi (Figure 6D; Figure S9). The primary divergence was localized between spongy mesophyll cells and defense‐responsive mesophyll cells. Notably, the proportion of defense‐responsive mesophyll cells increased markedly at 24 hpi following *P. polysora* infection (Figure 6D,E).

These results indicate that defense‐responsive mesophyll cells constitute a crucial component of the maize immune response to *P. polysora* infection. GO functional enrichment analysis of the DEGs within this subtype further showed that they are primarily involved in response to stress, abscisic acid‐mediated signaling pathway, and response to reactive oxygen species (Figure 6F). Among the DEGs associated with cellular response to stress, most were upregulated at 24 hpi (Figure 6G). Single‐cell data indicated that *Zm00001eb411490* (*ZmCIP1*, *Cytokinin‐inducible protease1*) was significantly upregulated in mesophyll cells at 24 hpi (Figure 6H). Spatial transcriptomic data corroborated this finding, highlighting its potential important role in the defense response (Figure 6I,J).

### 2.7 Cell‐cell Communication Reveals the Infection Process from Epidermal Cells to Mesophyll Cells

**FIGURE 7:**
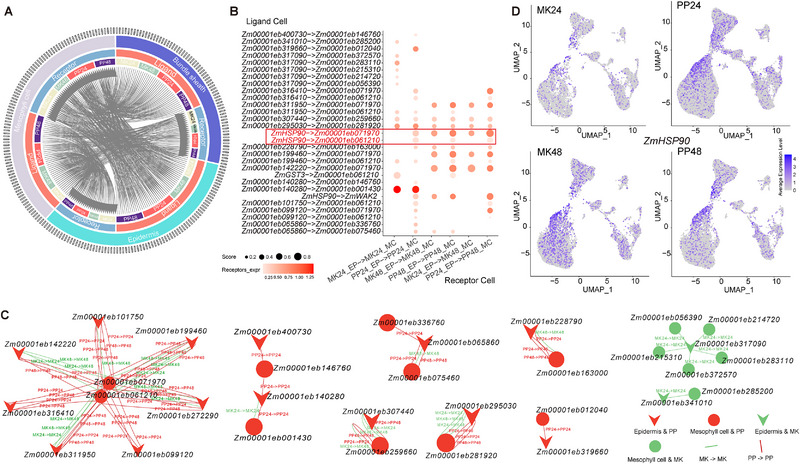
Cell‐cell communication reveals the process from epidermal cells to mesophyll cells. (A) Chord diagram showing the connections between CCC‐related genes in epidermal cells, mesophyll cells, and bundle sheath cells. The outer circle represents genes, the middle circle represents tissue types, and the innermost circle represents ligands and receptors, along with time. (B) Analysis of shared and specific CCC characteristics between in epidermal and mesophyll cells observed in snRNA‐seq data. Columns represent different communication directions for the same cell type, while rows indicate the corresponding communication direction between genes. Points size reflects the communication intensity score, and points color represents the expression level of the receptor genes. (C) Communication network generated from CCC in part B. Green represents control samples, while red indicates treated samples, with the shapes of the points signifying different tissue types and treatments. (D) Expression levels of the *ZmHSP90* gene obtained via snRNA‐seq in the treatment and control groups 24 and 48 hpi.

In the plant immune responses, coordinated interactions among diverse cell types are crucial for mediating defense against pathogen infection [19], with molecules such as hormones, growth factors, chemokines, cytokines, and neurotransmitters serving as ligands to facilitate intercellular communication, known as cell‐cell communication (CCC) [42]. Expression profiles were thus derived using snRNA‐seq and stRNA‐seq to identify the key genes involved in cell communication within critical leaf tissues, using data from the PlantPhoneDB database [43]. The analysis revealed significant genetic interactions among the epidermal, mesophyll, and bundle sheath cells (Figure 7A). Previous research has highlighted the essential roles that epidermal and mesophyll cells play in the defense of maize against *P. polysora* [15, 16]. Thus, the gene communication between mesophyll and epidermal cells was further analyzed using the snRNA‐seq expression profile, with results indicating specific cell interactions during *P. polysora* infection in maize (Figure 7B,C). For instance, the snRNA‐seq data demonstrated that the epidermal cell gene *Zm00001eb101750* (*ZmHSP90, Heat shock protein 90*) interacts with the mesophyll cell gene *Zm00001eb071970* (*ZmWAK2, Wall‐associated kinase 2*) during *P. polysora* infection (Figure 7B,C). Meanwhile, this study identified specific CCC in *P. polysora* infection in maize, revealing that *Zm00001eb272290* (*ZmHSP90*, a Heat Shock Protein 90 gene belonging to the same family as *Zm00001eb101750*) interacts with *Zm00001eb061210*, forming a cell‐type‐specific CCC predominantly detected in mesophyll and epidermal cells (Figure 7B). Furthermore, expression analysis demonstrated that *ZmHSP90* (*Zm00001eb272290*) exhibits a significant transcriptional upregulation 24 hpi during *P. polysora* infection, whereas its expression level remains unchanged 48 hpi (Figure 7D). Similarly, the stRNA‐seq datasets were processed with an identical analytical pipeline; however, the limited number of high‐confidence genes captured in these libraries severely constrained the detection of mesophyll–epidermis gene–gene communication pairs, yielding only a minimal set of interactions (Figure S10A). Of particular interest, a comprehensive comparative analysis revealed that the epidermal cell‐derived gene *Zm00001eb142220* (*ZmGST3*, *Glutathione Transferase 3*) demonstrates conserved interaction with the mesophyll cell‐expressed gene *Zm00001eb061210*, with this specific intercellular communication event being consistently identified across both snRNA‐seq and stRNA‐seq datasets (Figure S10B). Additionally, the stRNA‐seq data highlighted the high levels of expression of these two genes 24 hpi and 48 hpi with *P. polysora* (Figure S10C,D). Collectively, these findings emphasize the critical role of cell communication in the defense of maize against *P. polysora* infection.

### 2.8 Identification of Key Genes Involved in Maize Defense against *P. polysora*

**FIGURE 8:**
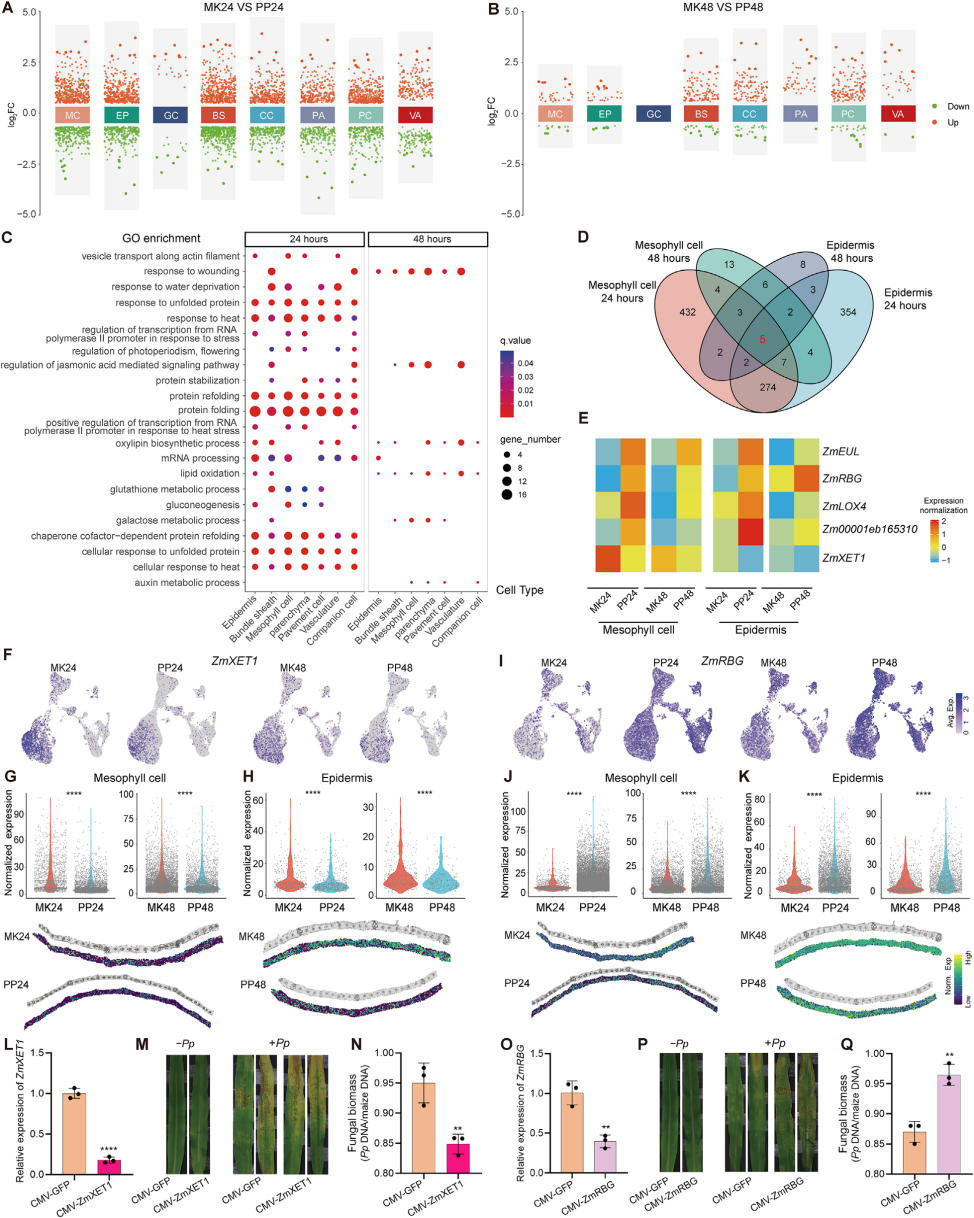
Key genes involved in the defense of maize against *P. polysora*. (A) Volcano plot of DEGs in different maize leaf cell types infected with *P. polysora* 24 hpi. Each column represents a specific leaf tissue, with the Y‐axis representing log_2_FC and each point representing a gene. Red points indicate upregulated genes, and green points indicate downregulated genes. Selection criteria: |log_2_FC| > 0.5 and *p*.value < 0.05. (B) Volcano plot of DEGs in different maize cell types infected with *P. polysora* 48 hpi. (C) Bubble plot of GO enrichment for differentially expressed gene sets across various plant tissues. Points size reflects the number of genes associated with each GO term, while the color represents the p‐value. All displayed GO terms have a *p* value of < 0.05. (D) Venn diagram showing the number of DEGs in mesophyll and epidermal cells under different treatments. The red number represents the count of shared key genes across both time points and cell types. (E) Relative expression heatmap of key genes in different tissues and time points, with color indicating TPM normalization. (F) Expression levels of the *ZmXET1* gene in treatment and control groups obtained via snRNA‐seq. (G–H) Upper panels: Expression levels of the *ZmXET1* gene across different samples obtained via stRNA‐seq. Lower panels: Corresponding spatial localization of *ZmXET1* expression from stRNA‐seq. Statistical significance was assessed by Student's *t*‐test: ****p < 0.0001, *n* = 2512 (MK24), 6092 (PP24), 6408 (MK48), 3261 (PP48) in mesophyll cells, and 455 (MK24), 663 (PP24), 478 (MK48), 326 (PP48) in epidermis cells. (I) Expression levels of the *ZmRBG* gene in treatment and control groups obtained via snRNA‐seq. (J–K) Upper panels: Expression levels of the *ZmRBG* gene across different samples obtained via stRNA‐seq. Lower panels: Corresponding spatial localization of *ZmRBG* expression from stRNA‐seq. Statistical significance was assessed by Student's *t*‐test: ****p < 0.0001, *n* = 1113 (MK24), 14127 (PP24), 4963 (MK48), 5000 (PP48) in mesophyll cells, and 755 (MK24), 4678 (PP24), 1165 (MK48), 1898 (PP48) in epidermis cells. (L) Analysis of the expression levels of the *ZmXET1* gene in CMV‐*ZmXET1* silenced plants and the control. Statistical analysis was performed using Student's *t*‐test: *** *p* < 0.001, *n* = 3. (m) Symptoms of CMV‐*ZmXET1* silenced maize leaves challenged with *P. polysora*. (N) Relative biomass assessment following fungal infection with *P. polysora* in CMV‐*ZmXET1*‐silenced maize leaves. Statistical analysis was performed using Student's *t*‐test: ** *p* < 0.01, *n* = 3. (O) Analysis of the expression levels of the *ZmRBG* gene in transiently silenced maize plants. Differences between groups were assessed using Student's *t*‐test: *p < 0.05, *n* = 3. (P) Symptoms development of *P. polysora* on CMV: *ZmRBG*‐silenced maize leaves. (Q) Relative biomass assessment of *P. polysora* following fungal inoculation in CMV‐*ZmRBG* silenced seedlings. Statistical analysis was performed using Student's *t*‐test: ** *p* < 0.01, *n* = 3.

To identify the key regulatory genes involved in the defense of maize against *P. polysora*, snRNA‐seq and stRNA‐seq were employed to determine the DEGs involved in the maize defense process. Initially, snRNA‐seq was utilized to identify DEGs across various cell types at different time points post‐infection, with the results indicating that the expression of a substantial number of genes changed in the maize leaves 24 h after infection, particularly in the mesophyll, epidermal, and bundle sheath cells (Figure 8A; Figure S11A,B). In contrast, the number of DEGs in guard cells was unexpectedly low (Figure 8A), which may be attributed to the relative abundance of other cell types identified in the snRNA‐seq analysis. Furthermore, the number of DEGs in each cell type significantly decreased 48 hpi, with no DEGs detected in guard cells as compared to the 24‐h time point (Figure 8B; Figure S11C,D). These findings suggest that the early phase of *P. polysora* infection in maize leaves represents the most complex stage of defense activation. Thus, to explore the functional roles of the DEGs, GO enrichment analysis was conducted for each cell type, with results revealing the substantial activation of functional pathways across all cell types 24 hpi, including protein folding, protein stability, mRNA processing, and chaperone cofactor‐dependent protein refolding (Figure 8C). In stark contrast, many functional pathways were not enriched 48 hpi (Figure 8C). At this later time point, the main functional responses observed were the response to wounding, regulation of the JA‐mediated signaling pathway, oxylipin biosynthesis, and lipid oxidation (Figure 8C), indicating the involvement of the JA‐mediated signaling pathway during *P. polysora* infection in maize leaves. Given that the hyphae of *P. polysora* invade maize leaves through the stomata or epidermal cells and absorb nutrients via the mesophyll cells, the study focused on uncovering key genes by identifying shared DEGs in the mesophyll and epidermal cells at both the 24 and 48 hpi. The results revealed five core genes common to both cell types at these points (Figure 8D). Of these, five genes: *Zm00001eb123630* (*ZmEUL*, *E3 ubiquitin‐protein ligase*), *Zm00001eb217560* (*ZmRBG*, *RNA‐binding glycine‐rich protein*), *Zm00001eb054050* (*ZmLOX4*, *lipoxygenase 4*), and *Zm00001eb165310* were upregulated following *P. polysora* infection, while *Zm00001eb226470* (*ZmXET1*, *xyloglucan endotransglycosylase homolog* 1) was downregulated (Figure 8E). The snRNA‐seq analysis revealed that *ZmXET1* was significantly downregulated in both the PP24 and PP48 samples (Figure 8F). Correspondingly, stRNA‐seq data indicated that the expression level of *ZmXET1* was significantly reduced in both mesophyll and epidermal cells following *P. polysora* infection (Figure 8G,H). Analysis of snRNA‐seq data showed that *ZmRBG* was significantly upregulated in both the PP24 and PP48 samples (Figure 8I), and a similar trend was confirmed by stRNA‐seq (Figure 8J,K). Similarly, the expression patterns of the other three genes, as assessed by both snRNA‐seq and stRNA‐seq, exhibited significant changes after *P. polysora* infection (Figure S12).

Based on these findings, phenotypic analysis of the relevant genes was then performed using virus‐induced gene silencing (VIGS). The maize materials developed in the study exhibited an approximately 70% reduction in *ZmXET1* gene expression (Figure 8L), and following inoculation with *P. polysora*, the VIGS‐silenced plants (CMV‐*ZmXET1*) displayed milder disease symptoms as compared to the control (Figure 8M). Further analysis showed a significant reduction in the pathogen biomass within the silenced plants, suggesting that *ZmXET1* negatively regulates the defense of maize against *P. polysora* (Figure 8N). Similarly, VIGS‐silenced *ZmRBG* plants exhibited reduced expression levels (Figure 8O). Unlike the control, CMV‐*ZmRBG* plants presented exacerbated disease symptoms post‐inoculation (Figure 8P), along with increased pathogen biomass (Figure 8Q). To address the functional relevance of our single‐cell findings at the cellular level, histological phenotypes of epidermal and mesophyll cells in the VIGS‐silenced lines following *P. polysora* inoculation were examined. Safranin O and fast green staining revealed that *ZmRBG*‐silenced plants exhibited a certain degree of structural compromise in both epidermal and mesophyll cells. After inoculation with *P. polysora*, these cells sustained more severe damage compared to those in the CMV‐GFP control. Conversely, epidermal and mesophyll cells in *ZmXET1*‐silenced leaves showed relatively milder impairment post‐infection (Figure S13A). Fungal colonization patterns across different cell types were further evaluated using WGA staining. Consistent with the quantified pathogen biomass, the most extensive WGA‐stained area and the strongest fluorescence signal were observed in CMV‐*ZmRBG* leaves, indicating the highest fungal proliferation. This extensive colonization aligns with the severe cellular damage observed by histology. An intermediate level was shown by the CMV‐GFP control, while the smallest fungal area and weakest signal were displayed by the CMV‐*ZmXET1* line‐a pattern corroborating the milder cellular impairment in this genotype (Figure S13B). Thus, the WGA‐based quantification not only reflects pathogen burden but also provides independent support for the cell integrity differences revealed by safranin O and fast green staining. These results collectively indicate that both genes contribute to maize resistance to *P. polysora* through distinct regulatory mechanisms, while also validating the reliability of the multi‐omics data.

## 3 Discussion

### 3.1 An Integrated Spatial‐temporal Atlas Uncovers a Novel Cellular Defense Paradigm in Maize

SCR can significantly impair photosynthesis, leading to reduced kernel fill, lower yields, and diminished corn stalk quality [4, 6]. This study successfully establishes the first integrated single‐nucleus and spatial transcriptomic atlas of the maize defense against *P. polysora*, providing a stereoscopic view of the early immune landscape in a resistant maize genotype (Figure 2A) [44]. By combining snRNA‐seq and stRNA‐seq 24 and 48 hpi, the previously inaccessible spatial and cellular heterogeneity of maize leaves responding to the fungal challenge were resolved (Figure 2A). Crucially, this approach enabled us to move beyond bulk transcriptomics and identify not only key hub genes but also their operational context within the specific cell types and tissue domains, with the mesophyll and epidermal cells particularly highlighted as important defense tissues. Functional validation of central genes such as *ZmXET1* and *ZmRBG* further supports the roles of these tissues within this spatially organized immune network. Both were predicted by our multi‐omics network, underscoring the power of this approach in pinpointing causal genes within specific cellular contexts.

### 3.2 Cell Type‐specific Immune Strategies and Coordination

Our data revealed that maize employs a division of labor among several different cell types to mount an effective defense (Figure 4A). While epidermal and mesophyll cells showed attenuated early immune signaling, possibly explaining the initial success of fungal penetration, inner tissue layers, especially the bundle sheath and vascular parenchyma, exhibited strong defense activation programs (Figure 4A), including ROS burst amplification and phytohormone signaling (Figure 5C, E,G). Such spatial compartmentalization suggests a strategy in which internal cells serve as a second line of defense, activating mechanisms that restrict pathogen spread even after the initial infection occurs. This is reminiscent of the “vascular immunity” reported in Arabidopsis and rice but with maize‐specific features, including a stronger emphasis on cell wall remodeling than on phytoalexin production [45]. By targeting the specific cell types and pathways within this network, strategies can be developed to strengthen the innate immunity of maize, leading to improved agricultural practices and crop resilience [46].

### 3.3 Limitations and Inferences of Fungal Presence

One notable limitation in this study was the low representation of fungal transcripts in the sequencing data. This is likely attributable to multiple factors: the obligate biotrophic nature of *P. polysora*, its tight regulation by the host microenvironment, the early sampling time points, and the inherent difficulty in controlling the precise inoculation sites. Although the spore suspension solution was applied uniformly, the actual infection foci were stochastic and early fungal biomass was likely below the detection limit of snRNA‐seq and stRNA‐seq. Consequently, we could not reliably analyze fungal gene expression or quantify pathogen growth at the cellular level. Future studies would benefit from investigating later time points (72–96 hpi), the targeted laser microdissection of infection sites, or complementary imaging‐based methods to correlate the host transcriptomic responses with fungal localization while also considering the effect of maize cell death at the infection site on the transcriptional data.

### 3.4 Assessing improved strategies for exploring the molecular mechanisms of maize's interaction with *P. polysora*

While the genes we identified are associated with the defense response in a resistant line, their differential expression could potentially be observed in susceptible lines as well, albeit possibly with different kinetics or amplitudes. The definitive classification of these genes as resistance markers would require future comparative transcriptomic studies involving both resistant and susceptible isolines. We thus emphasize that our study provides a valuable foundation for the better understanding of candidate genes and pathways, and that the specificity for resistance of these genes and pathways must be validated through further functional studies with contrasting genotypes.

Whereas stRNA‐seq provided a valuable spatial context, its current resolution remains insufficient to fully distinguish adjacent cell types or subcellular fungal structures. In addition, the presence of the fungus and the associated host cell death may have influenced the nuclear recovery and RNA quality in heavily infected zones, and the detection of transcripts from deeper pathogenic structures was hindered, affecting the resolution. Technical challenges related to sample preparation and data integration are also likely; thus, further refinement is required. Future research could benefit from integrating single‐cell and spatial transcriptomic technologies with advanced imaging techniques, such as confocal microscopy, to enhance our insight of the infection dynamics. Additionally, combining these transcriptomic approaches with proteomics and metabolomics would offer a more holistic understanding of the interaction between maize and *P. polysora*. Combining these with genetic perturbations, such as the silencing of *ZmXET1* or *ZmRBG*, will help validate the proposed mechanisms and uncover causal relationships within the maize immune network.

### 3.5 Agricultural Implications and Applications

Despite these limitations, our multiomic atlas provides a foundational resource for understanding the spatial immunity in maize. This study identified not only key resistant gene sets but also the role of these gene sets in the defense of maize against *P. polysora*, and is expected to provide a basis for identifying the mechanism of core genes involved in the immune process and enable a more targeted approach to improve the resistance to SCR. The results suggest that precision breeding or synthetic biology strategies aimed at enhancing the responses of specific cells could offer more effective and yield‐stable resistance. Finally, the inferred role of longitudinal gradient defense, akin to that reported in rice, may further inspire strategies for designing crops with spatially optimized immunity [24].

## 4 Experimental Section

### 4.1 Plant Materials and *P. Polysora* Inoculation and Collection

PP.CN1.0 *P. polysora* isolates were collected from infected maize leaves (Zhengdan 958 cultivar) at the experimental station of Henan Academy of Agricultural Sciences using sterilized toothpicks and transferred to 2 mL centrifuge tubes. A homogeneous spore suspension was then prepared in a 0.01% Tween solution (with ddH_2_O dilution), yielding a solution with pale‐yellow to light‐brown coloration. For manual inoculation, 20–40 µL aliquots were gently applied along the leaf base‐to‐tip axis using pipette tips, ensuring no epidermal damage. Inoculated leaves were then mist‐irrigated and maintained in darkness at 25°C within a humidity‐controlled chamber for 12–24 h and transferred to standard photoperiod conditions post‐incubation.

Maize plants of the resistant inbred line Qi319 were cultivated at the Henan Academy of Agricultural Sciences in an illuminated incubator under controlled conditions of a 14‐h light/10‐h dark photoperiod and a temperature of 20°C–25°C. When the maize reached the V3 growth stage, *P.polysora* spores were suspended in a 0.01% Tween 20 solution to a concentration of approximately 1×10^6^ spores/ml, and the spore suspension evenly applied to all leaf surfaces using a small spray bottle. A parallel control group (Mock, MK) was established with a suspension solution lacking *P. polysora* (Tween 20 dilutied to 0.01% in ddH_2_O). Inoculated leaves, spanning from the leaf collar to the tip, were then collected 24 hpi and 48 hpi for further analysis.

### 4.2 Fluorescence WGA Process

Plant tissue samples were collected and subjected to decolorization using a bleaching solution (ice acetic acid: anhydrous ethanol = 1:1) until the leaves became translucent and devoid of chlorophyll. The decolorized tissues were then treated with chloral hydrate for 1–2 days and the obtained samples rinsed twice in 50% ethanol for 15 min, followed by 1–2 10‐min rinses in distilled water. The samples were then transferred to a 1 m potassium hydroxide solution and placed in a boiling water bath for 20–30 min to soften the leaf tissues, facilitating the penetration of fluorescent dyes into the mesophyll cells. The samples were then soaked in 50 mM Tri‐HCl (pH 7.0–7.4) for 30 min, stained with 20 µg/mL of WGA staining solution in the dark for over 10 min, and rinsed 2–3 times with distilled water for 10 min. Observations and photography were conducted under the green fluorescent protein (GFP) channel.

### 4.3 Histological Analysis by Safranin O‐Fast Green Staining

Maize leaf samples were fixed, dehydrated, and embedded in paraffin. Sections of 4 µm thickness were cut using a Leica RM2016 rotary microtome, mounted on slides, and dried. Following deparaffinization and rehydration, sections were stained with Safranin O for 2 h, briefly rinsed, differentiated in a graded ethanol series (50%, 70%, 80%), and counterstained with Fast Green (6–20 s). After dehydration and clearing in xylene, sections were mounted with neutral gum. Stained sections were examined and imaged under a Nikon ECLIPSE E100 upright optical microscope (Nikon, Tokyo, Japan) equipped with a Nikon DS‐U3 imaging system. Subsequent image analysis was performed using saiviewer software (ServiceBio, Wuhan, China). Lignified cell walls appear red and cellulose‐rich walls green, allowing for evaluation of tissue morphology and cellular integrity.

### 4.4 Nuclei Isolation, Single‐Cell Library Preparation

Leaf segments were immersed in cryoprotectant (5 mL/sample: 1.5 mL sterile water, 2.5 mL glycerol, 1 mL FBS) for 5 min on ice. The cryoprotectant was then removed by tilting the tube at a 45° angle and gently aspirating the majority of the solution with a pipette tip placed near the surface. After brief centrifugation at 100 × *g* and 4 °C, the remaining solution was carefully withdrawn from the bottom of the tube using a 1 mL sterile syringe and discarded, and the resulting samples flash‐frozen in liquid nitrogen. Nuclear isolation began with tissue homogenization using a glass Dounce grinder (Sigma, Cat # D8938) in 2 mL of ice‐cold EZ lysis buffer, with 25 strokes using pestles A/B. After adding 3 mL of buffer, the samples were incubated on ice for 5 min and centrifuged at 500 *g* for 5 min at 4°C. Washed nuclei pellets were treated with nuclei suspension buffer [NSB; 1× PBS, 0.01% BSA, 0.1% RNase inhibitor (Clontech, Cat. no. 2313A)], filtered through 35 µm strainers (Corning‐Falcon, Cat. #. 352235), and quantified for snRNA‐seq.

### 4.5 Single‐Nucleus Sequencing

Single nucleus suspensions were prepared in PBS/0.04% BSA processed using the Chromium Next GEM Single Cell 3' Reagent Kits v3.1 (10× Genomics) on the Chromium Controller. GEMs were generated by chip loading (Chromium Next GEM Chip G) following the manufacturer's protocols. After nuclear lysis and RNA barcoding through GEM‐based reverse transcription, cDNA libraries were constructed and quality‐controlled using Qubit 4.0 and Agilent 2100. Sequencing was then performed on an Illumina NovaSeq 6000, with >50 000 PE150 reads per nucleus (Biomarker Technologies Corporation, BMKGENE, Beijing, China).

### 4.6 Processing Raw Data and Assay From snRNA‐seq of 10× Genomics

Sequencing data were aligned using 10× Cell Ranger v7.0 (STAR aligner‐engineered) against the maize B73 reference genome (v5.0) [47], and gene expression quantification performed using unique molecular identifiers (UMIs) for cell barcode‐gene pairing. Nuclear‐containing cell barcodes were filtered through the intrinsic quality control pipeline in Cell Ranger v7.0, and subsequent analyses (clustering, cell type annotation, and differential expression profiling) carried out only on validated nuclei populations using Seurat v4.0.1.

### 4.7 Dimensionality Reduction

To facilitate unsupervised clustering and cell type identification, principal component analysis (PCA) was applied for dimensionality reduction on the combined tissue sample set. For data visualization, Seurat was used to further reduce the dimensionality of all nuclei and *t*‐SNE employed to project the cells into 2D space. The procedure involved: 1) Calculating the gene expression values using the Log‐Normalize method from the “Normalization” function in Seurat; 2) Performing PCA on the normalized expression values and selecting the top principal components for clustering and *t*‐SNE analysis; and 3) Identifying clusters using the weighted Shared Nearest Neighbor (SNN) graph‐based clustering method. Marker genes for each cluster were determined using the “bimod” (Likelihood‐ratio test) with default parameters in the FindAllMarkers function in Seurat (v4.0.1) [48]. The top 10 genes were selected as marker genes by filtering the FindMarkers results for a Fold Change of >1.5 and FDR < 0.1.

### 4.8 Clustering and Cell Type Identification

Cluster partitioning was determined through the Louvain algorithm‐based community detection operating on nearest‐neighbor graphs derived from the PCA space. Cellular ontology classification was conducted using a neural network classifier that was trained to map single‐nucleus transcriptomic profiles onto standardized cell types. The annotation pipeline integrated automated SingleR (v1.4.1) predictions leveraging seven reference datasets, with manual refinement used to incorporate sub‐ontology cellular state specifications. Final cell type validation was performed by cross‐referencing against the PlantCellMarker (https://www.tobaccodb.org/pcmdb/homePage), PlantscRNAdb (http://ibi.zju.edu.cn/plantscrnadb/) and PsctH (http://jinlab.hzau.edu.cn/PsctH/) repositories for phylogenetic consistency [33, 34, 35].

### 4.9 Identification of Immune‐Related Genes

Protein sequences of the primary transcripts of maize B73 genome were used to identify resistance genes using DRAGO2 (v2) (http://prgdb.org/prgdb4/) [49]. Previous research has found that, typical genes involved in PTI include RLPs and RLKs [38], with RLPs consisting primarily of an extracellular domain capable of binding ligands and a transmembrane domain and RLKs generally containing an additional kinase domain as compared to RLPs. [38] Genes in ETI are Typical R genes, which are classified based on their domains into NL genes (containing NBS and LRR domains) and CNL genes (containing CC, NBS, and LRR domains) [39]. It is generally considered that monocot plants, such as maize, do not possess TIR domains. Regarding the genes associated with the JA and SA pathways, this study drew upon relevant work by Wang et al. in rice [24].

### 4.10 Trajectory Inference

Monocle 3 (https://cole‐trapnell‐lab.github.io/monocle3/) facilitates the reconstruction of cellular trajectories from single‐cell RNA‐sequencing data through a streamlined workflow [50, 51]. It begins with data normalization, feature selection, and dimensionality reduction via UMAP. Cells are then partitioned into distinct trajectories using graph‐based clustering and a partitioned approximate graph abstraction (PAGA) to identify significantly connected communities. A principal graph is learned within each partition to model detailed transition paths, incorporating landmark sampling for scalability. Pseudotime is computed by projecting cells onto this graph and measuring geodesic distance from a user‐specified root node. Optionally, genes with trajectory‐dependent expression patterns can be identified using spatial autocorrelation statistics such as Moran's I, enabling the discovery of genes associated with cellular differentiation.

### 4.11 Frozen Embedded Tissue for Spatial Transcriptomics RNA Sequencing

Tissue samples were collected from maize leaves and cut into small fragments (6.8 mm^2^) for experimentation. The tissue fragments were then snap‐frozen in isopentane pre‐chilled with liquid nitrogen, combined with an optimal cutting temperature (OCT) compound (SAKURA, Cat#: 4583), and stored at −80°C until use.

### 4.12 Slide Preparation

Spatial transcriptomics slides featured 1–8 identical capture areas, each measuring 6.8 × 6.8 mm and containing 2 000 000 barcoded primer spots (BMKMANU, ST03002). The primers, secured at the 5' end, included a cleavage site, T7 promoter region, a partial read1 Illumina handle, a unique spatial barcode, a unique molecular identifier (UMI), and Poly(dT)VN, and were arranged in a centered hexagonal grid, with each spot having a diameter of 2.5 µm and a center spacing of 4.8 µm from its six neighboring spots. Tissue samples were then sectioned into 10 µm thick slices using a pre‐cooled cryostat and placed onto chilled BMKMANU Tissue Optimization and Gene Expression Slides, and stored at −80°C until use.

### 4.13 Fixation, Staining, and Imaging

Tissue sections from the previous step were pre‐incubated at 37°C for 1 min, fixed with 3.7% formaldehyde (Sigma‐Aldrich) in PBS (Medicago) for 30 min, and rinsed with PBS. Sequential staining was performed with Mayer's hematoxylin (Dako) for 4 min, followed by a 30‐s dip in bluing buffer and 30 s in eosin (Sigma‐Aldrich) that was diluted at 1:5 in Tris‐buffer (0.45 M Tris/0.5M acetic acid, pH 6.0). Samples were rinsed in nuclease‐free water performed between steps. The slides were then mounted in 85% glycerol (Merck Millipore), imaged at 20× magnification using a MetaSystems whole‐slide scanner, and stitched using VSlide software. After imaging, the coverslips were removed, and pre‐permeabilization performed with collagenase (0.5 U/mL, ThermoFisher) and BSA (0.2 mg/mL, NEB) in HBSS at 37°C for 20 min, followed by 0.1× SSC washes and pepsin digestion (0.1% in 0.1M HCl, Sigma‐Aldrich) at 37°C for 7 min, and termination with 0.1× SSC.

### 4.14 The Optimal Permeabilization Time

After obtaining permeabilized tissue sections, the optimal time point was selected based on fluorescence intensity. The fluorescence signal directly reflects the amount of mRNA released from the tissue, which is captured on the array. According to the experimental results of BMKGENE (Biomarker Technologies Corporation, Beijing, China), the optimal outcome is achieved when the mRNA is captured as close as possible to its original cellular location. Diffuse fluorescence or signal extending beyond cellular boundaries typically indicates over‐permeabilization, leading to mRNA diffusion. If two time points yield similar fluorescence performance, the longer time is generally recommended as the optimal permeabilization condition to ensure sufficient mRNA release.

### 4.15 Spot Visualization, Image Alignment, and BSTMatrix Analysis

Fluorescent probe hybridization enabled primer spot visualization via Metafer imaging. BSTMatrix (v2.0) was used to co‐register the fluorescence signals with the corresponding bright‐field histology, utilizing integrated tissue recognition algorithms to resolve spatially occluded spots. Libraries (4 nM) were sequenced on an Illumina NovaSeq 6000 (paired‐end). For all samples, raw sequencing data were subjected to quality control using fastp (v1.0.1) with specified parameters (‐Q ‐y ‐g ‐Y 10 ‐l 60 ‐b 150 ‐B 150) to generate clean data. The clean FASTQ files were then analyzed with BSTMatrix 2.0, along with manually aligned histology images. The resulting data were then aligned to the maize B73 reference genome (v5.0) using the STAR aligner (v2.5.1b). Processed alignment outputs were imported into R via the Seurat package (v4.0.1), spatial spots with over 30% mitochondrial gene content or fewer than 300 detected genes filtered out. Genes detected in fewer than five spatial spots were discarded. Spots exhibiting tissue folding were also removed. Finally, raw counts were normalized using the SCTransform function in Seurat with the parameter assay = “spatial”.

### 4.16 Correlation Analysis Between stRNA‐seq and snRNA‐seq

Multimodal Intersection Analysis (MIA) was used to assess the relationship between the data obtained using stRNA‐seq and snRNA‐seq. MIA is a multimodal integration method that annotates cell subpopulations in spatial transcriptomic data by detecting significant overlaps between the signature marker genes identified in single‐cell subclusters and those enriched in spatial transcriptomic regions.

### 4.17 Co‐Expression Network Analysis

To elucidate the key functional modules that underlie the maize defense against *P. polysora*, network topology analysis, a suite of methods for quantifying the structural and connectivity properties of constructed gene co‐expression networks based on nodes and edges, was employed to identify critical hub genes. Specifically, hdWGCNA (v0.4.07) was used to detect co‐expression networks within high‐dimensional transcriptomic data [52]. The hdWGCNA approach enables the identification of highly interconnected gene modules and evaluates their associations across different samples, while facilitating the construction of cell type‐specific co‐expression networks. To ensure scale‐free topology of the resulting networks, parameter screening was performed using the TestSoftPowers function. By executing the ConstructNetwork function with default parameters, a soft‐thresholding power of 3 was identified as optimal for the construction of robust co‐expression networks. The resulting WGCNA dendrogram was visualized using the PlotDendrogram function, and harmonized module eigengenes were projected onto dimensionality reduction plots using the ModuleFeaturePlot function. Functional enrichment analysis of the identified modules was conducted using the R package clusterProfiler (v4.2.2) with significance thresholds of *p* value < 0.01 and *q* value < 0.05 [53].

### 4.18 Differential Gene Expression Analysis

The FindAllMarkers function in the Seurat package was employed for statistical testing and analysis. Specifically, genes with raw counts below 3 across all sampled cells were excluded, and only genes expressed in more than five cells within at least one sample were retained for further analysis. The DESeq2 (v1.48.2) pipeline was applied to identify DEGs between treated and control groups, while also accounting for the gene expression patterns across different cell types to facilitate subsequent categorization. In line with previously established criteria, genes with a *p* value of < 0.05 and |log_2_FC| > 0.58 were defined as DEGs for the respective cell types [54, 55].

### 4.19 Cell Communication Analysis

Data from the maize species in the public database PlantPhoneDB (https://jasonxu.shinyapps.io/PlantPhoneDB/) were used to construct ligand‐receptor pairs across different samples, with the aim of exploring the CCC processes during *P. polysora* infection of maize leaves [43]. Cell–cell interactions were inferred based on the expression of ligand‐receptor pairs in different cell types, and significant ligand‐receptor pairs (*p*‐value < 0.05) indicative of active cell communication were identified through statistical significance evaluation.

### 4.20 RNA in Situ Hybridization

Maize leaves were collected for preparation of the transverse ultrathin sections. The plant material was fixed in 4% paraformaldehyde (Sigma) in 0.1 m phosphate buffer (pH 7.2) for 16 h at 4°C, then dehydrated by ethanol series and embedded in Paraplast Plus (Sigma). Sections (7–10 µm) were then cut using a Leica RM 2135 microtome (Leica, Germany), collected on xylene‐coated SuperFrost Plus slides (Menzel‐Glazer), and the slides deparaffinized and treated with 10 µg/mL proteinase K before subjection to in situ hybridization using previously described protocols [25]. The sequences of probes used for RNA hybridization are listed in Supplementary Table S8.

### 4.21 VIGS Assay

VIGS assays were conducted following a published protocol with minor modifications [56, 57]. The tripartite ZMBJ‐CMV genome was reconstituted using the pCMV101, pCMV201, and pCMV301 vectors to establish a VIGS platform targeting *ZmXET1* (*Zm00001eb226470*) and *ZmRBG* (*Zm00001eb217560*). Silencing fragments were designed via the SGN VIGS portal (https://vigs.solgenomics.net) with engineered primers (Table S9). The fragments were then cloned into pCMV201, resulting in pCMV201‐*ZmXET1* and pCMV201‐*ZmRBG* constructs. The recipient maize cultivar used in this study was the inbred line B73.

*Agrobacterium tumefaciens* GV3101 was transformed with the ternary plasmid system (pCMV101/pCMV201/pCMV301) using pCMV201‐*GFP* as a control. Bacterial suspensions (OD_600_ = 0.8) were incubated for 3 h prior to infiltration. Three‐week‐old *Nicotiana benthamiana* leaves (3rd–4th positions) were syringe‐infiltrated and maintained in controlled‐environment chambers, and infected leaf tissues (3–5 dpi) homogenized in ice‐cold 0.01M phosphate buffer (pH = 7.0) (1 mL/g tissue), centrifuged (4°C, 4500 rpm, 3 min), before applying the supernatants to B73 maize embryos via microinjection (15 µL/seed). Precision inoculation needles (60° insertion angle, 1–2 mm depth) were used to minimize embryo damage. Seeds were germinated in the dark at 25°C for 3 d on moistened filter paper, then transferred to soil and retained under 20°C/18°C (16 h‐light/8 h‐dark) conditions.

### 4.22 qRT‐PCR Analysis

Total RNA was isolated using Trizol reagent, and approximately 2 µg used for reverse transcription using cDNA synthesis with HiScript III RT Super Mix (+gDNA wiper) (R323‐01, Vazyme, China). qRT‐PCR was then performed on a QuantStudio 5 system (Thermo Fisher Scientific, USA) in a 25 µL reaction mixture containing 12.5 µL of LightCycler SYBR Green I Master Mix, 2 µL of diluted cDNA (1:5), 8.9 µL of distilled H_2_O, 0.8 µL of forward primer (10 mM) and 0.8 µL of reverse primer (10 mM). The primers used are listed in Supporting Information Table S9. Real‐time PCR data were analyzed by the comparative 2^−ΔΔCT^ method to quantify relative gene expression [58]. Three biological replications were performed on each sample, and three technical repeats of PCR analysis were conducted. The statistical significance was evaluated using Student's *t*‐test.

### 4.23 *P. polysora* Biomass Quantification

Standard curves were generated by cloning reference genes *ZmUbi* (maize) and *PpTub* (*P. polysora*) into pMD19‐T vectors. Serial plasmid dilutions (100 ng/µL, 10 ng/µL, 1 ng/µL, 10^−1^ ng/µL, 10^−2^ ng/µL, 10^−3^ ng/µL, 10^−4^ ng/µL) were used as templates for qRT‐PCR, with gene‐specific primers used to calibrate the cycle threshold (Ct)‐DNA concentration relationships. *P. polysora*‐inoculated CMV‐silenced and control plants were sampled 12–14 days post‐treatment for genomic DNA extraction, while qRT‐PCR analysis, using pathogen/host reference primers, was used for absolute DNA quantification. Relative biomass ratios (*P. polysora* DNA/Maize DNA) between the experimental and control groups were statistically compared. The relevant primers designed in this project are shown in Supplementary Table S9.

### 4.24 Complementary Methods

Plots were generated using R scripts. Box plots, stacked histograms, pie charts, bubble charts, and other graphs were using with ggplot2 v3.5.1 (https://ggplot2.tidyverse.org/). All heatmaps were generated using ComplexHeatmap v2.20.1 (https://jokergoo.github.io/ComplexHeatmap‐reference/book/index.html). The functional annotations for maize genes used in this study were retrieved from https://maizegdb.org/. For detailed functional annotation of the relevant genes, please refer to Supplementary Table S10.

### 4.25 Statistical Analysis

The preprocessing of snRNA‐seq and stRNA‐seq data in this study involved dimensionality reduction and normalization. Data from reproducible biological experiments are presented as mean ± standard deviation. VIGS experiments were designed with a sample size of six per group for statistical analysis. Significant differences were determined using Student's *t*‐test for two‐group comparisons or ordinary one‐way ANOVA followed by Tukey's HSD post hoc test for multiple‐group comparisons. Statistical significance was defined as *p* < 0.05. The snRNA‐seq analysis incorporated three biological replicates, while stRNA‐seq utilized four biological replicates. For differentially expressed gene identification and GO enrichment analysis, both *p* values and false discovery rate (FDR)‐adjusted *q*‐values were applied. All statistical analyses were performed using the R programming language.

## Author Contributions

Q.W., X.S., Y.S., and Z.C. contributed equally to this work. Y.C., C.D., and Y.S. conceived and designed the study. Q.W., X.S., Y.S., Z.C., Zi.C., S.H., Y.F., and W.Y. performed most of the experiments and data analysis. J.T. and Ho.L. provided valuable suggestions. H.L., M.Z., X.W. and J.Z. participated in some experiments. Q.W., Y.C., C.D. and Y.S. wrote the manuscript and revised the manuscript with feedback from all other authors. All authors agreed on the final version of the manuscript.

## Conflicts of Interest

The authors declare that they have no conflicts of interest.

## Supporting information




**Supporting File 1**: advs74678‐sup‐0001‐SuppMat.pdf.

**Supporting File 2**: advs74678‐sup‐0002‐Suppl Tables.xlsx.

## Data Availability

The raw data of snRNA and stRNA sequencing generated in this study can be obtained from the National Center for Biotechnology Information with accession nos. PRJNA1283969 and PRJNA1283968.
