# Supplementary material for: Integrated Single‐Cell and Spatial Transcriptomics Reveal Cell‐Type‐Specific Immune Regulatory Networks in Maize Responding to Southern Corn Rust
Source: Adv Sci (Weinh). 2026 Mar 16;13(27):e12295. doi: 10.1002/advs.202512295 (PMC13170197; doi:10.1002/advs.202512295)
Supplement: Supplementary file 1 — Supporting File 1: advs74678‐sup‐0001‐SuppMat.pdf. [file ADVS-13-e12295-s001.pdf]

Supporting Information

Integrated single-cell and spatial transcriptomics reveal cell-type-specific immune regulatory networks in maize responding to southern corn rust

Qiongqiong Wang, Xinyan Sun, Yingchao Sun, Zeqiang Cheng, Zixiang Cheng, Shengbo Han, Ying Feng, Wenbo Yang, Huimin Li, Meichen Zhu, Xiaoling Wu, Jinghua Zhang, Jihua Tang, Honglian Li, Yanyong Cao*, Canxing Duan*, and Yan Shi*

Supplementary Fig. 1

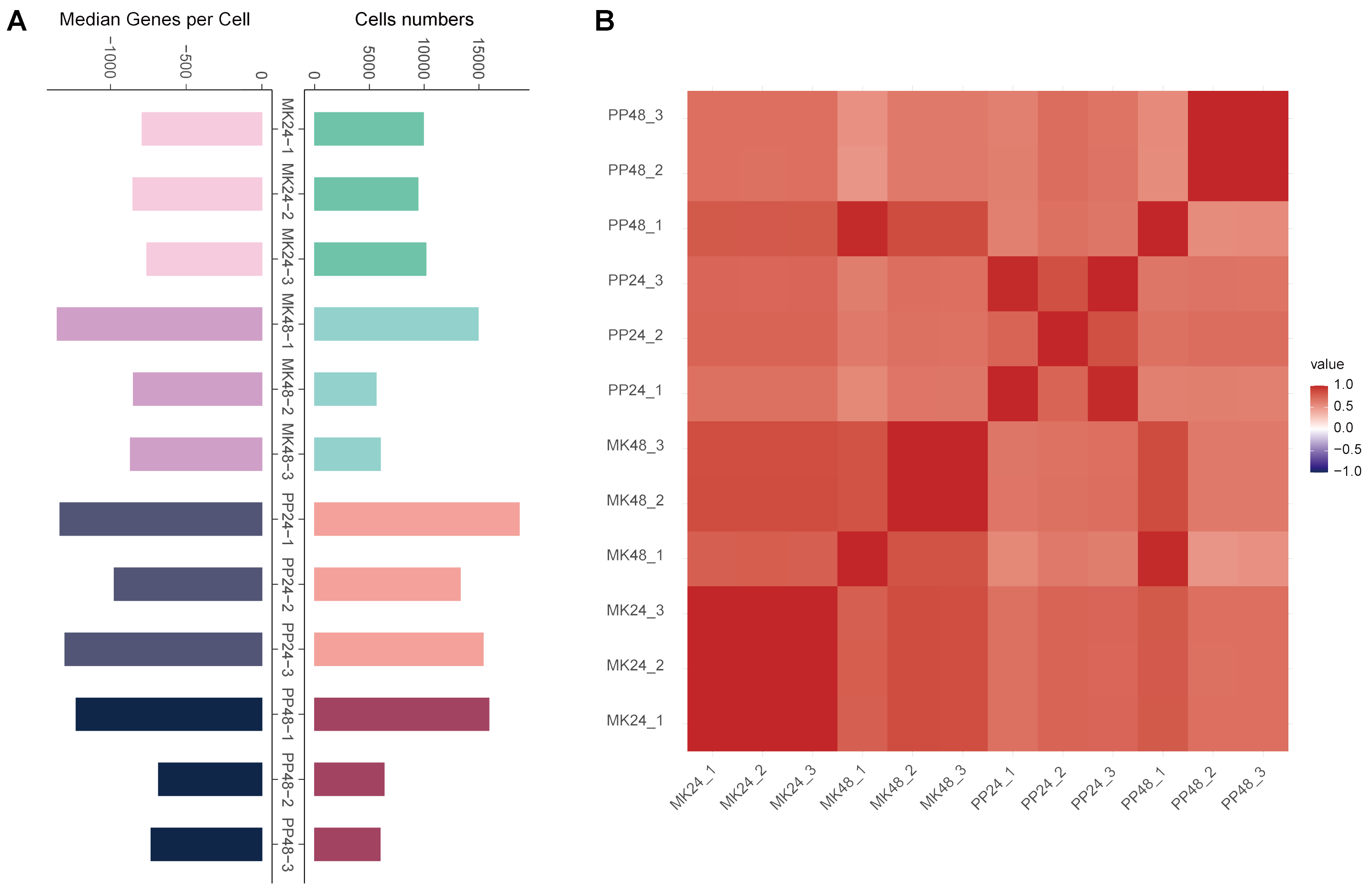

Supplementary Fig.1 Quality analysis of snRNA sequencing results. A) Histogram showing the distribution of median genes per cell and the number of cells for each snRNA sequencing sample. B) Correlation analysis between each sample of snRNA sequencing.

Supplementary Fig. 2

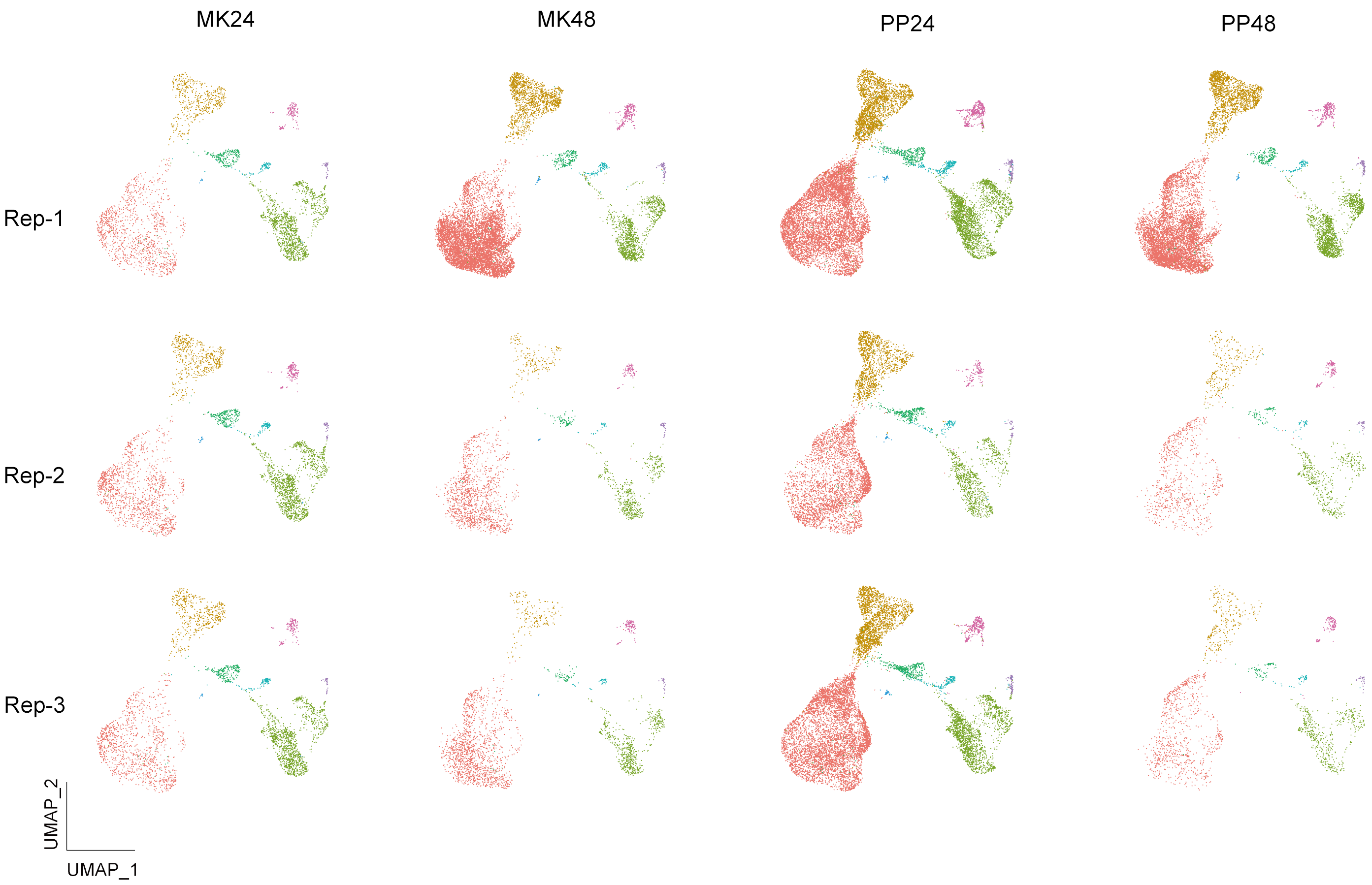

Supplementary Fig. 2 Mapping of snRNA-seq clustering across different samples.

Supplementary Fig. 3

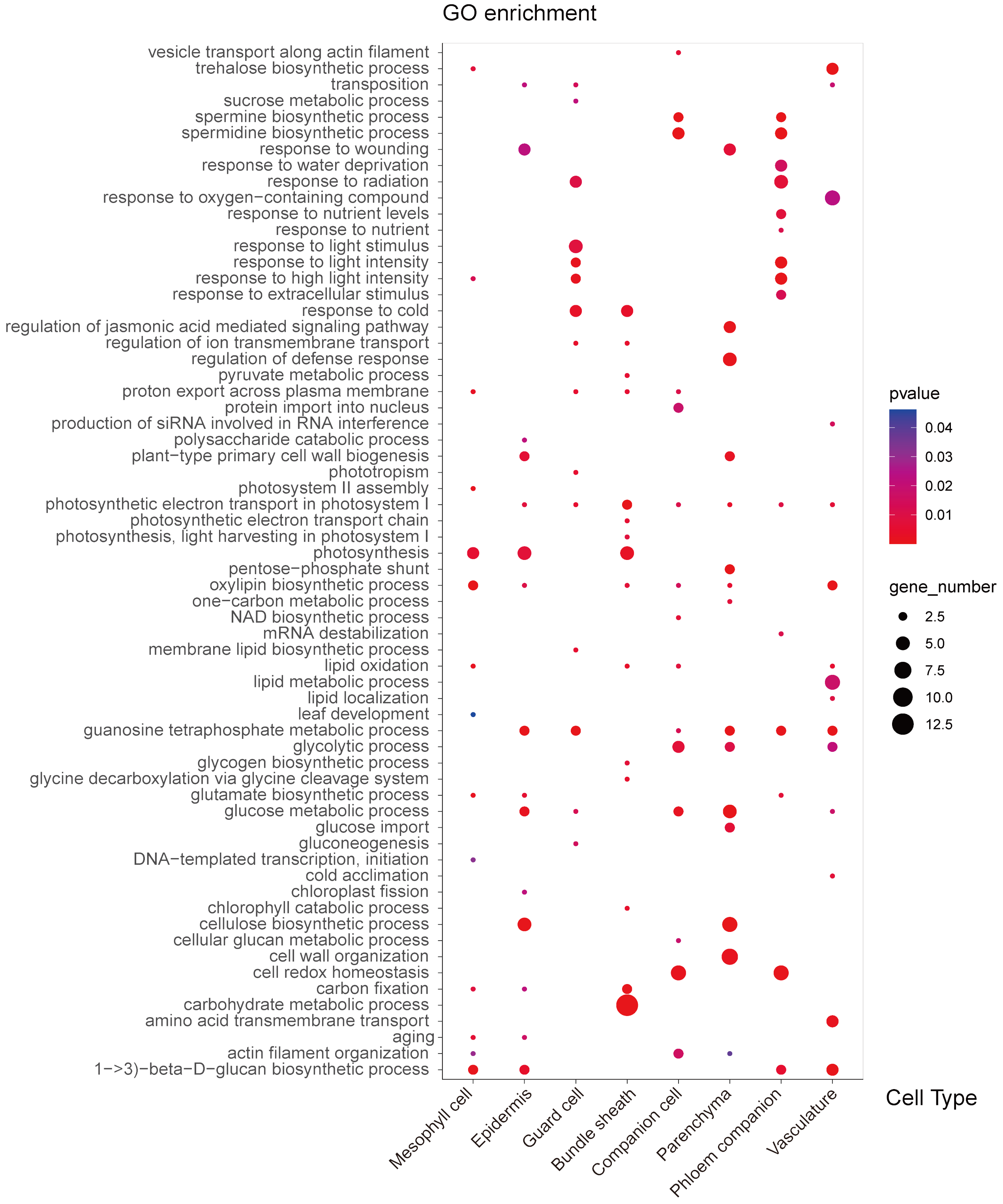

Supplementary Fig. 3 GO enrichment of expressed genes in different cell types in snRNA-seq.

Supplementary Fig. 4

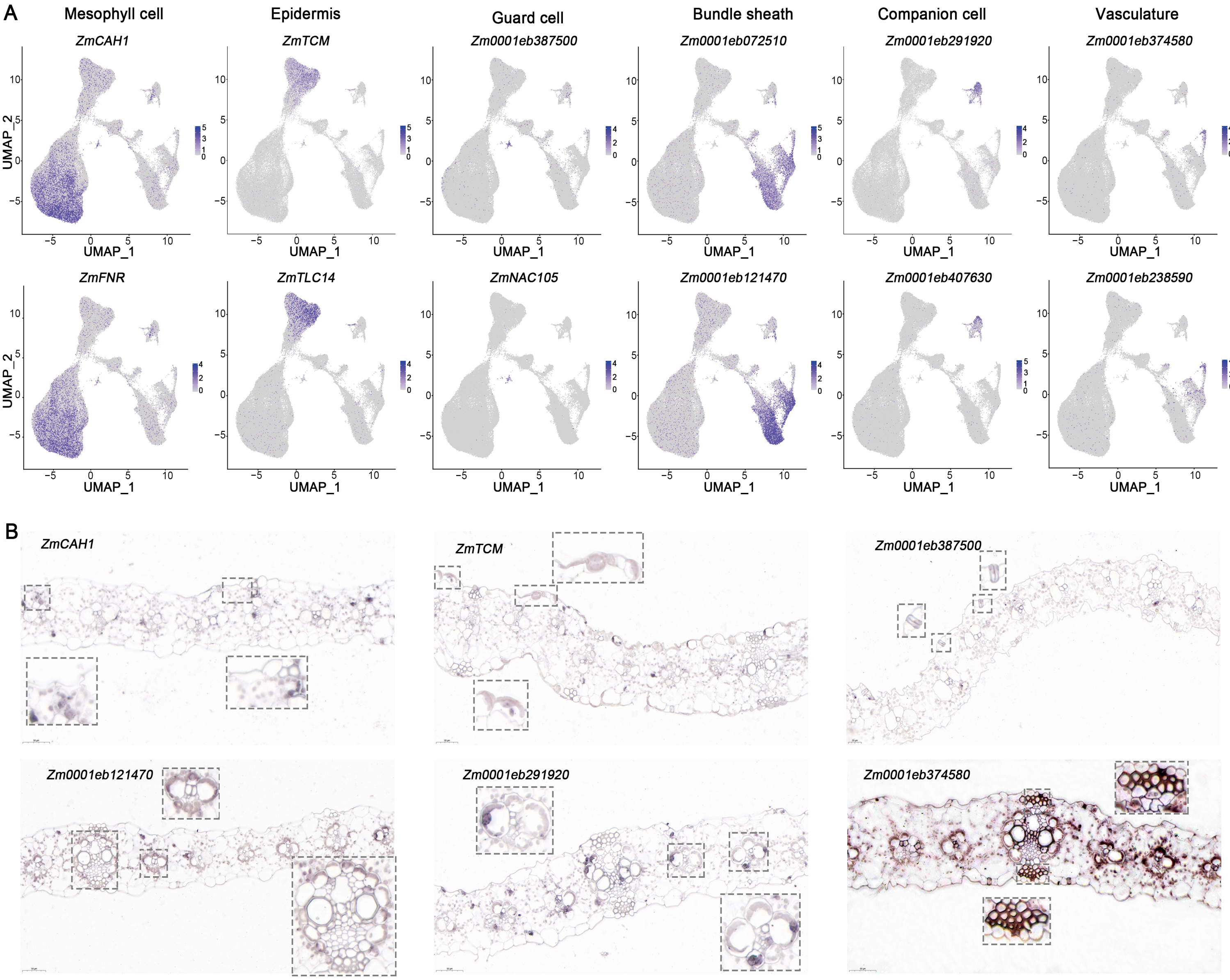

Supplementary Fig. 4 Identification of high-confidence Top2 marker genes in different tissue clusters. A) UMAP visualization of expression patterns of cell cluster-specific marker genes, and color represents their relative expression levels in these cell clusters. B) RNA *in situ* hybridization validation of representative cell type-specific marker genes for the putative cell types. Images within the dashed boxes show magnified views of the *in situ* hybridization signals.

Supplementary Fig. 5

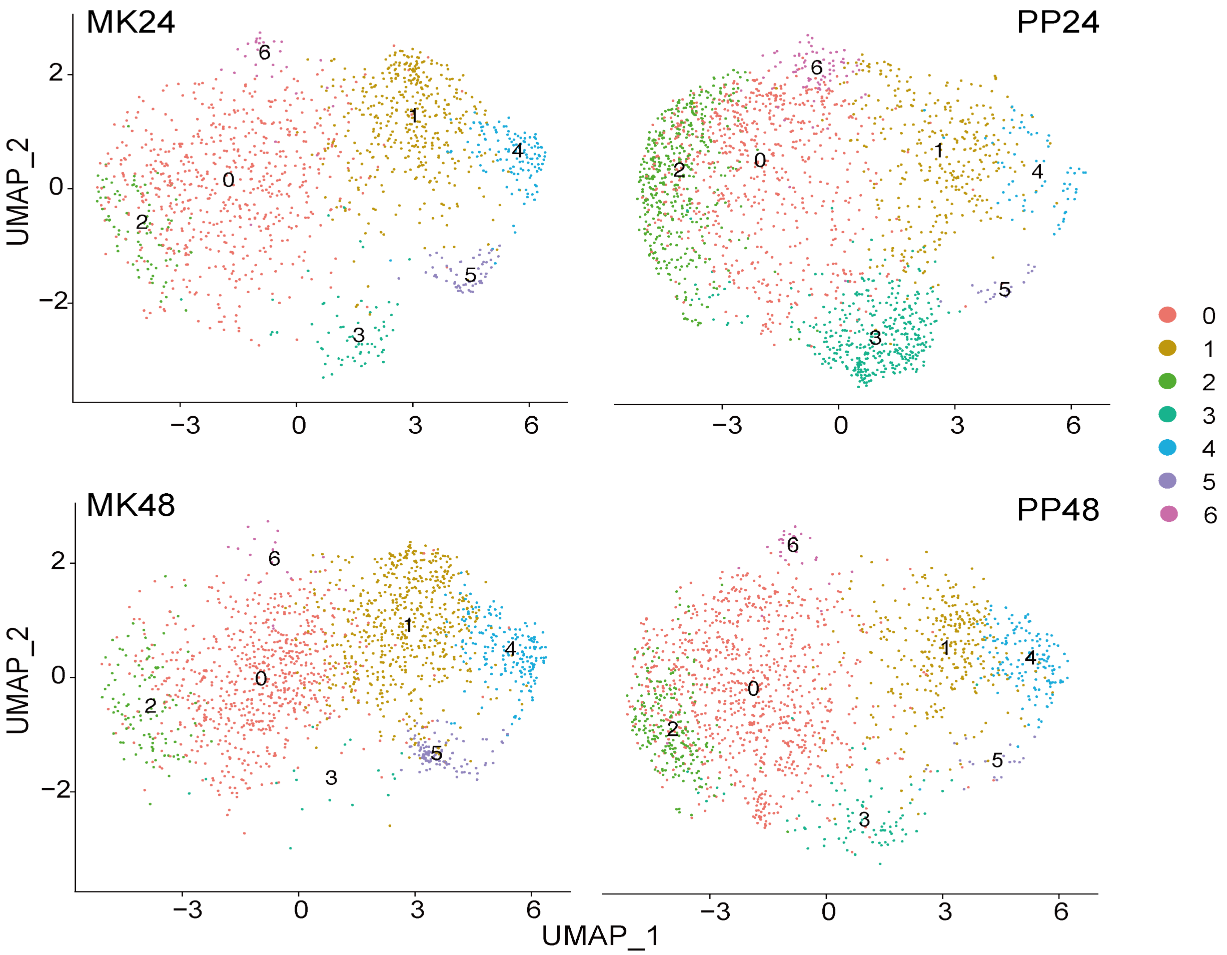

Supplementary Fig. 5 Dimensionality reduction and mapping of stRNA-seq in different samples of spatial transcriptomics.

Supplementary Fig. 6

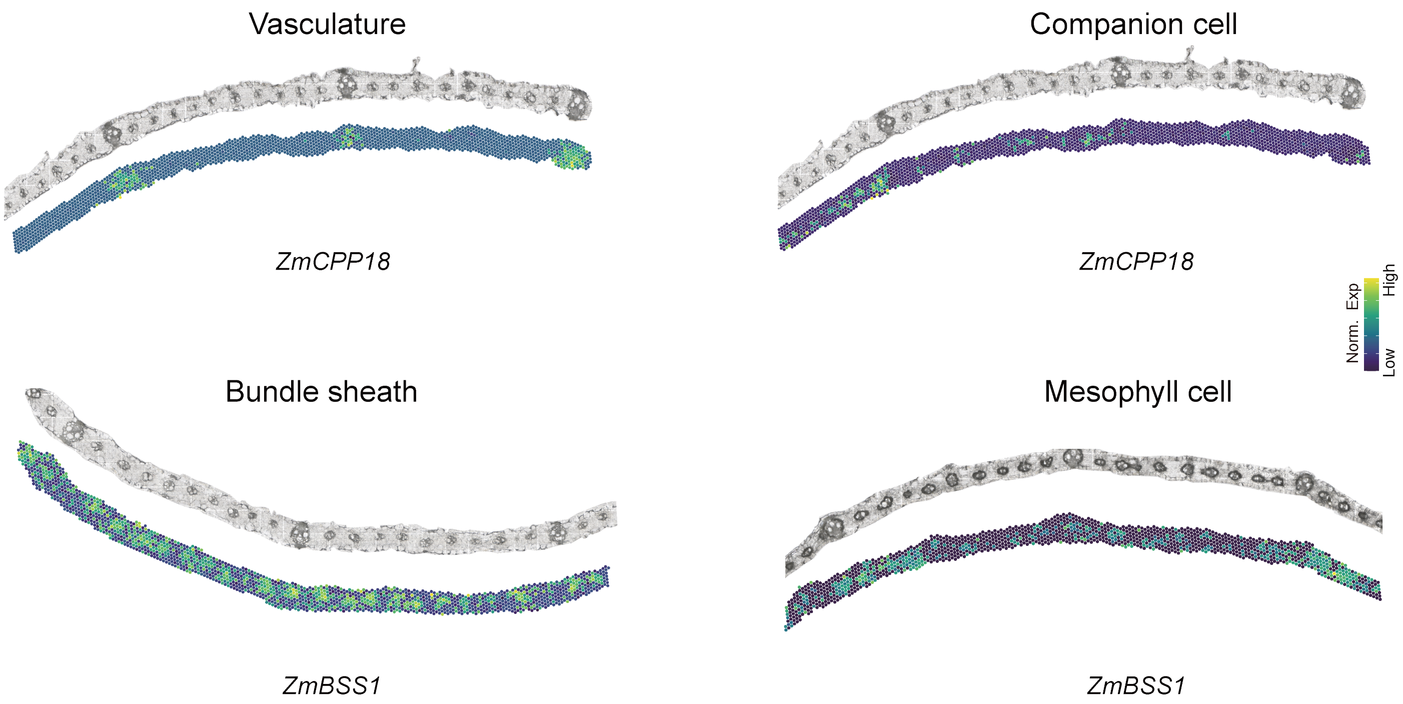

Supplementary Fig. 6 Expression of marker genes identified in stRNA-seq across different clusters. Norm. Exp., normalized expression.

Supplementary Fig. 7

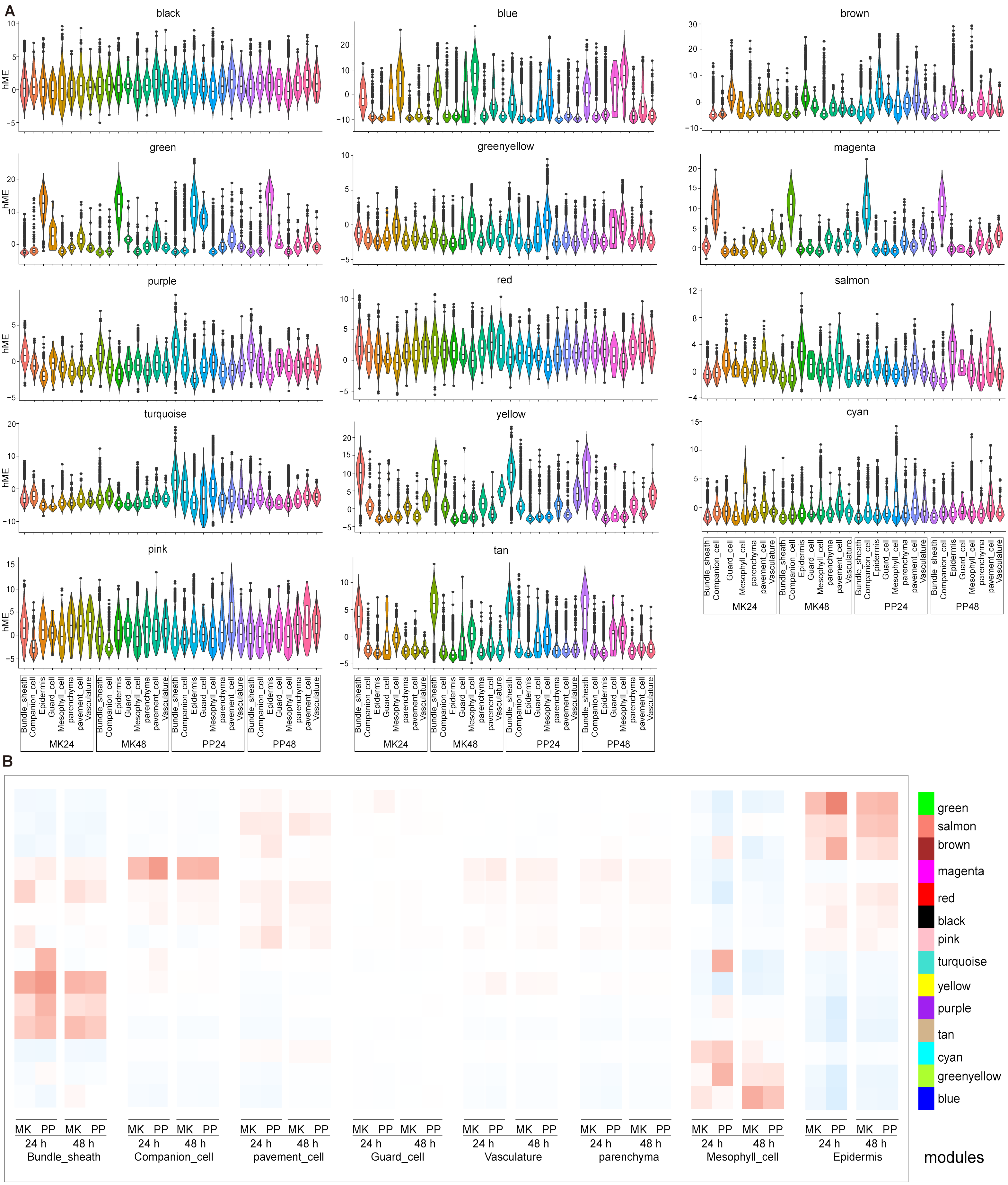

Supplementary Fig. 7 Co-expression modules and their correlation with different cell types. A) Expression levels of relevant genes in each module across different cell types. B) Correlation analysis of different modules and cell types under different treatments. Red indicates a positive correlation, while blue indicates a negative correlation.

Supplementary Fig. 8

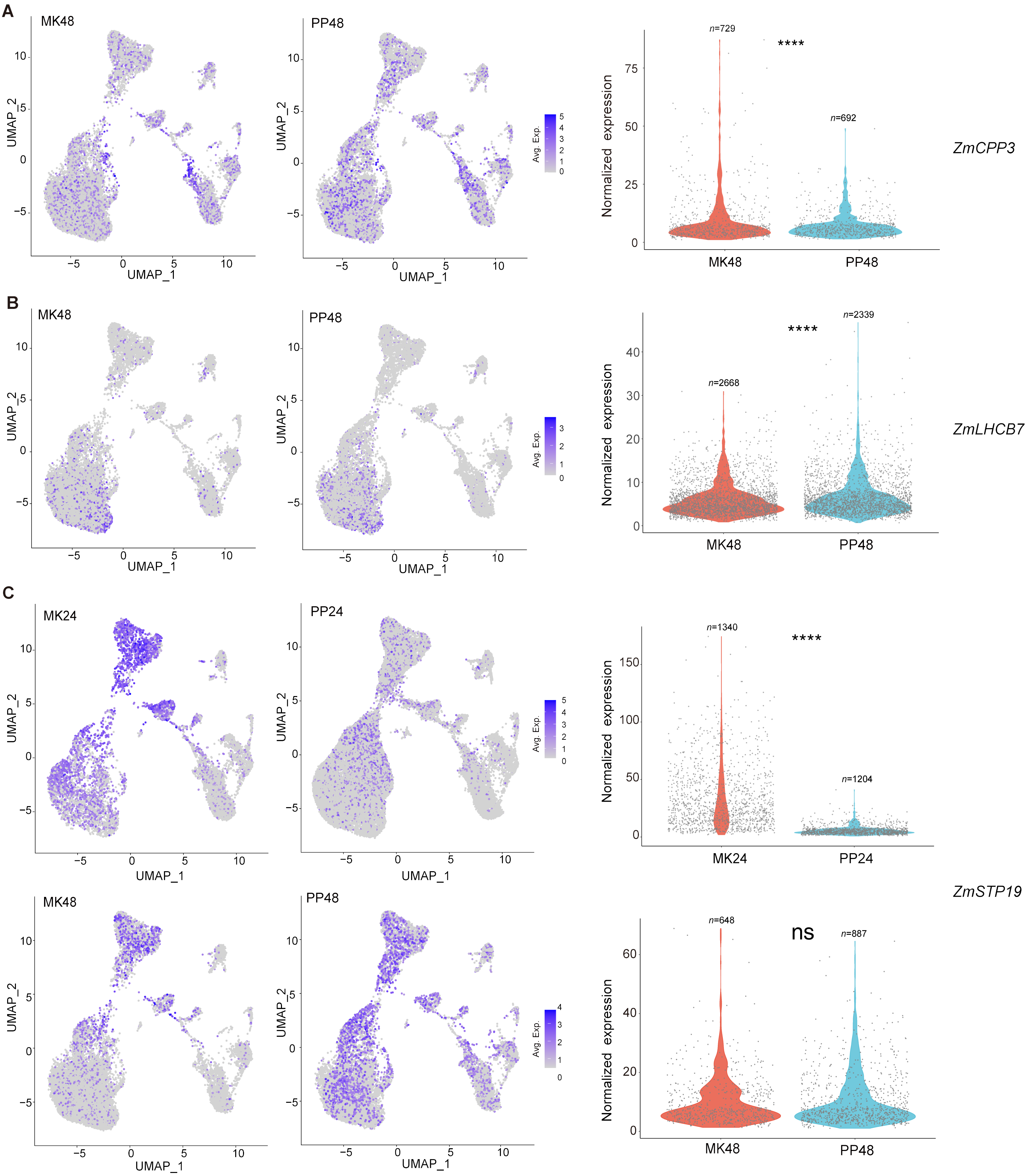

Supplementary Fig. 8 Expression levels of the three genes in snRNA-seq and stRNA-seq. A) Left panels: Expression of the *ZmCPP3* gene in different clustering locations obtained via snRNA-seq at 48 hours in treatment and control groups. Right panels: Violin plot showing the expression level of *ZmCPP3* in bundle sheath cells from stRNA-seq data;each point represents an individual cell. Statistical analysis was performed using Student's *t*-test: *****p* < 0.0001. B) Left panels: Expression of the *ZmLHCB7* gene in different clustering locations obtained via snRNA-seq at 48 hours in treatment and control groups. Right panels: Violin plot showing the expression level of *ZmLHCB7* in mesophyll cells from stRNA-seq data; each point represents an individual cell. Statistical analysis was performed using Student's *t*-test: *****p*< 0.0001. C) Left panels: Expression of the *ZmSTP19* gene in different clustering locations obtained via snRNA-seq at 24 and 48 hours in treatment and control groups. Right panels: Violin plot showing the expression level of *ZmSTP19* in epidermal cells from stRNA-seq data; each point represents an individual cell. Statistical analysis was performed using Student's *t*-test: *****p* < 0.0001. The numbers on the violin plots indicate the cell numbers (*n*).

Supplementary Fig. 9

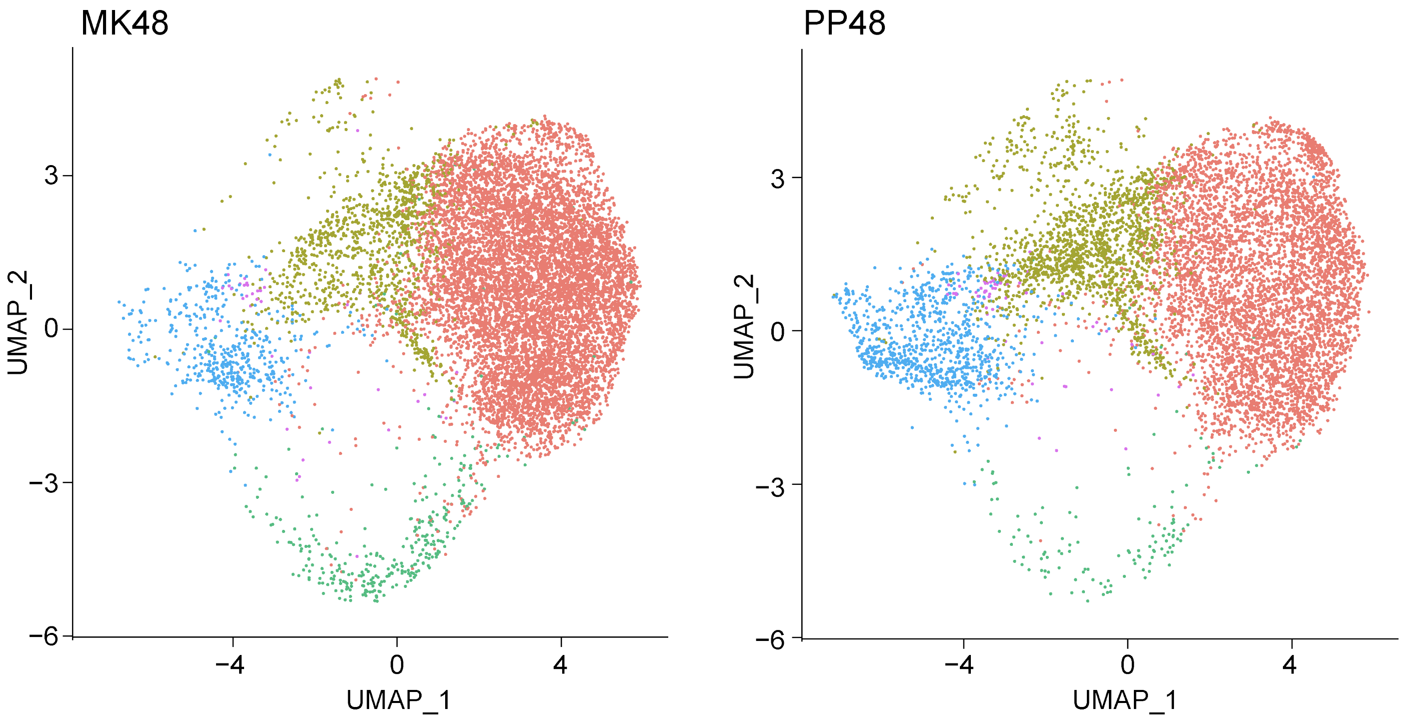

Supplementary Fig. 9 Divergence in differentiation trajectories of mesophyll cells across experimental conditions at 48 hpi.

Supplementary Fig. 10

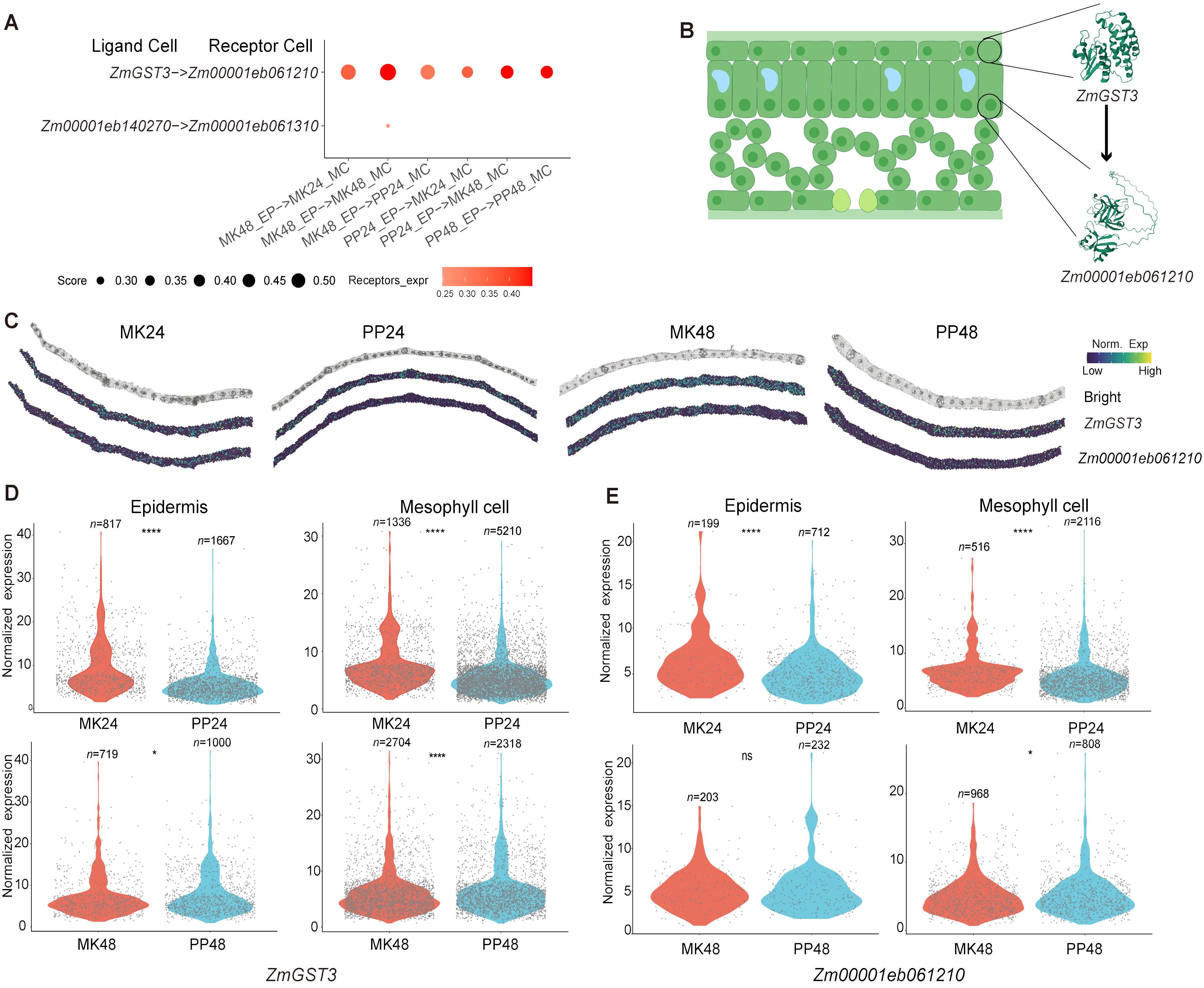

Supplementary Fig. 10 Cell-cell communication reveals the process from epidermal cells to mesophyll cells for stRNA. A) Analysis of shared and specific CCC characteristics in stRNA-seq data. B) Pair of shared CCC genes selected from snRNA-seq and stRNA-seq data: *ZmGST3* in epidermal cells and *Zm00001eb061210* in mesophyll cells. C) Expression levels and locations of *ZmGST3* and *Zm00001eb061210* obtained via stRNA-seq treatment and control groups 24 hpi and 48 hpi. Norm. Exp., noralized expression. D– E) Violin plot showing the expression level of *ZmGST3* and *Zm00001eb0612 10* in epidermal cells and mesophyll cells from stRNA-seq data; each point represents an individual cell. Statistical analy sis was performed using Student's *t*-test: ns, not significant; *$p < 0.05$, ****$p < 0.0001$. The numbers on the violin plots in dicate the cell numbers (*n*).

Supplementary Fig. 11

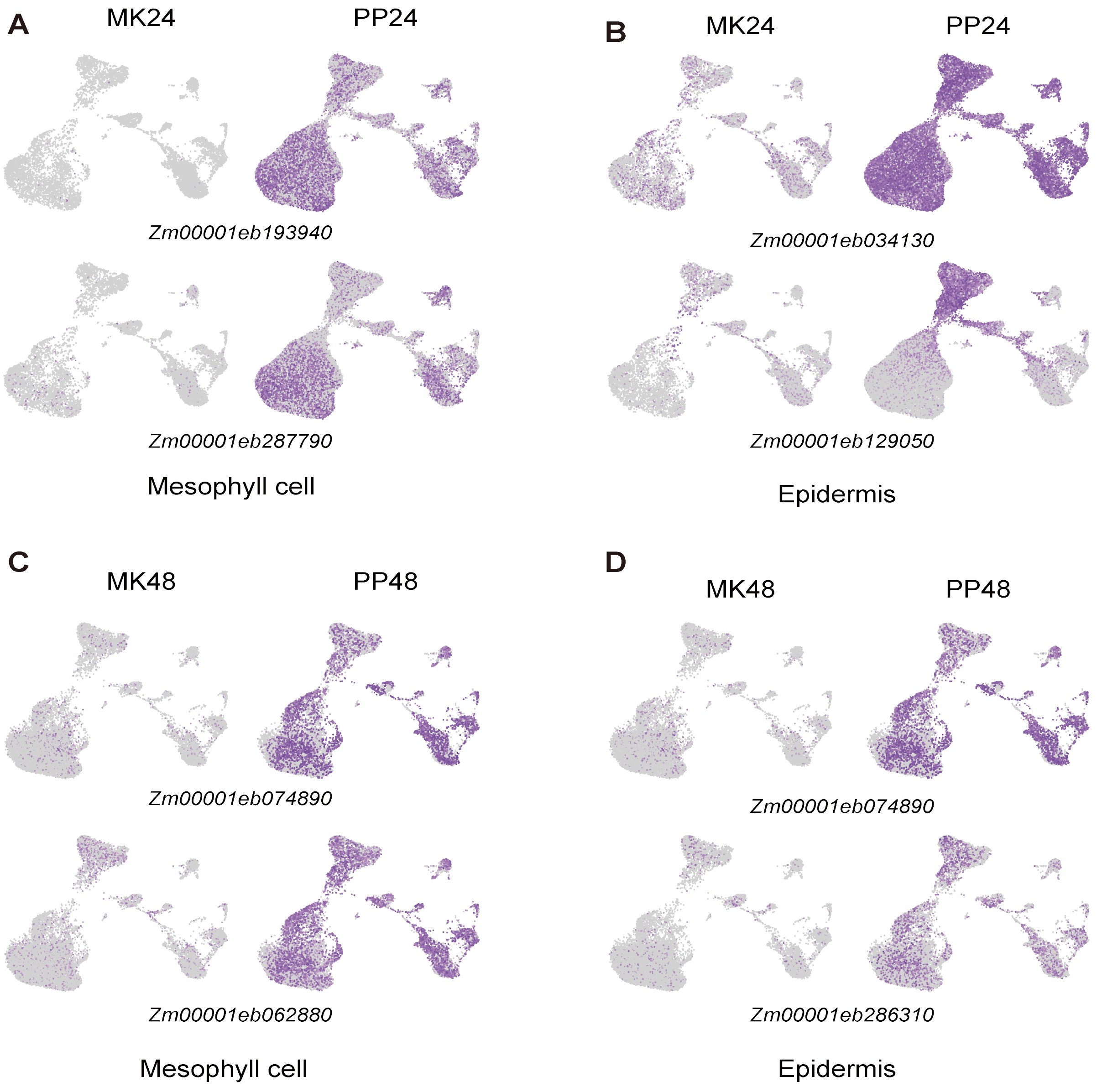

Supplementary Fig. 11 Expression levels of representative DEGs from different cell types in snRNA-seq. A) Representative DEGs in mesophyll cells at 24 hpi. B) Representative DEGs in epidermal cells at 24 hpi. C) Representative DEGs in mesophyll cells at 48 hpi. D) Representative DEGs in epidermal cells at 48 hpi.

Supplementary Fig. 12

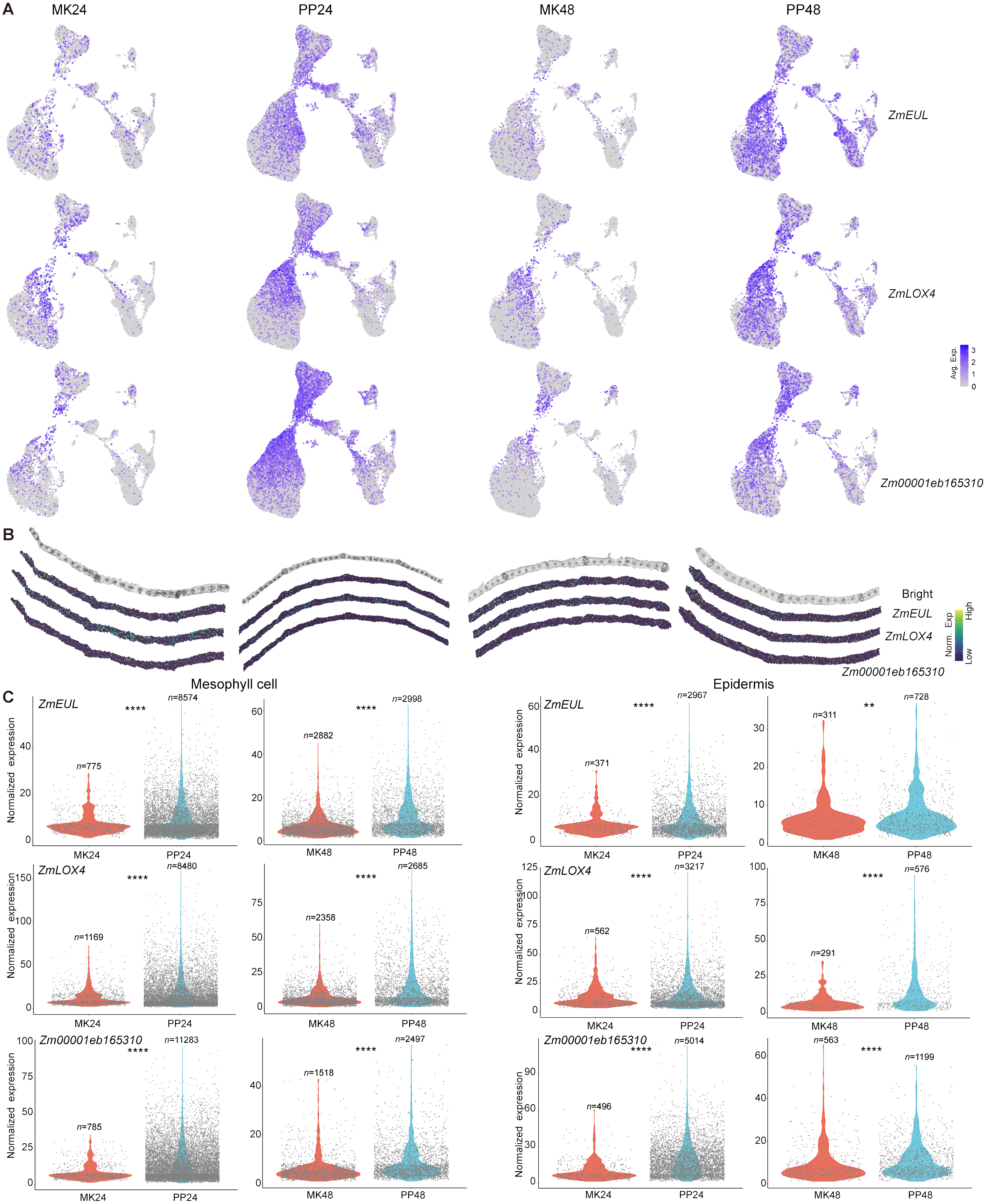

Supplementary Fig. 12. A) Expression of *ZmEUL*, *ZmLOX4*, and *Zm00001eb165310* in different clustering locations obtained using snRNA-seq 24 and 48 hpi in both treatment and control groups. B) Expression of *ZmEUL*, *ZmLOX4*, and *Zm00001eb165310* in different locations obtained via stRNA-seq at 24 hpi and 48 hpi in treatment and control groups.. C) Violin plot showing the expression level of *ZmEUL*, *ZmLOX4*, and *Zm00001eb165310* in mesophyll cells and epidermis from stRNA-seq data; each point represents an individual cell. Statistical analysis was performed using Student ' s *t*-test: ** $p < 0.01$, ****$p < 0.0001$. Value on the violin plots indicate the cell numbers (*n*).

Supplementary Fig. 13

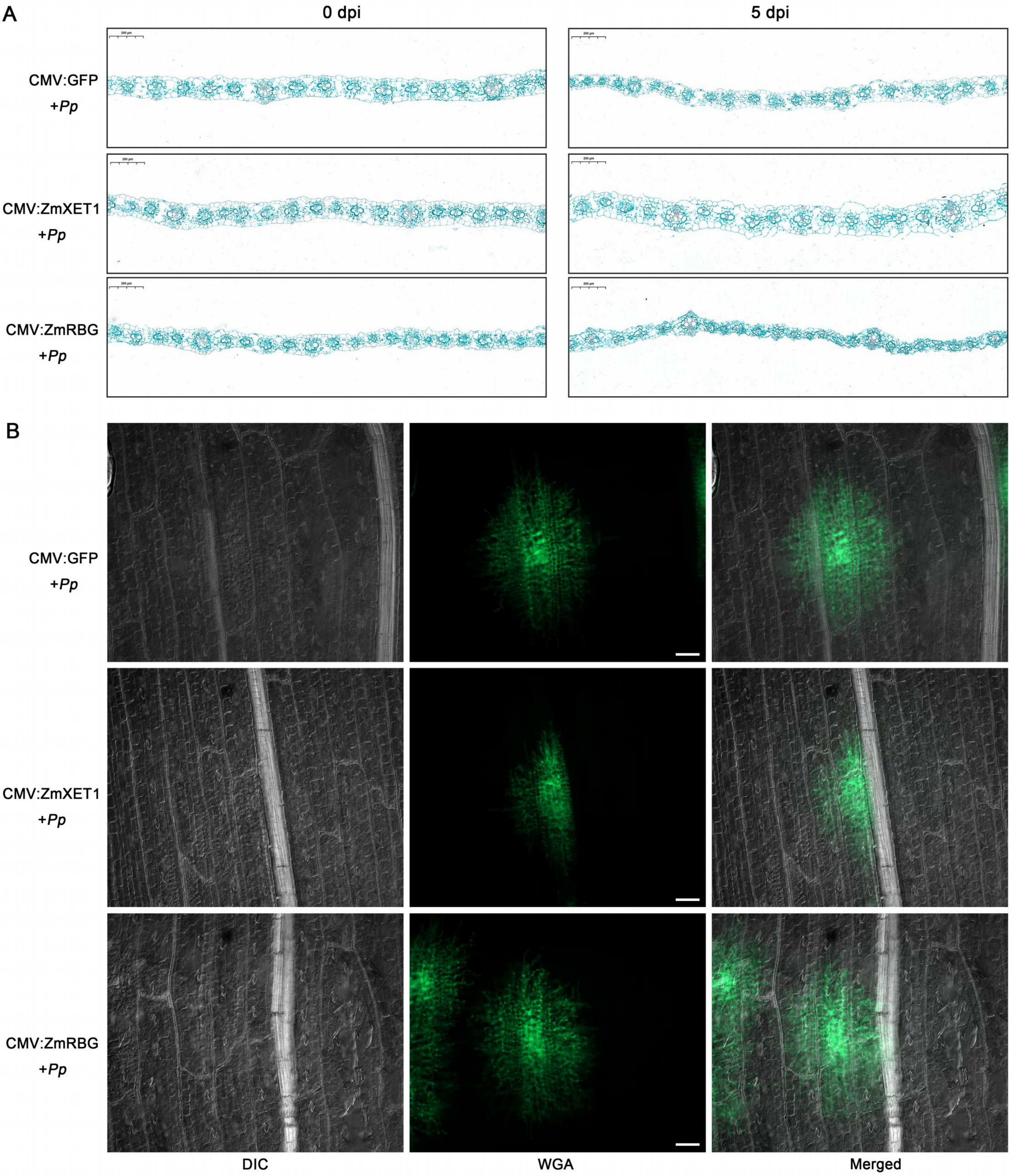

Supplementary Fig. 13 Histological and fungal colonization analysis of epidermal and mesophyll cells in VIGS-silenced maize leaves after *P. polysora* inoculation. A) Representative images of Safranin O and Fast Green staining in cross-sections of leaves from CMV-GFP (control), CMV-ZmRBG, and CMV-ZmXET1 plants at 0 dpi and 5 dpi with *P. polysora* inoculation. Lignified structures appear red and cellulose-rich walls green. Scale bars=200 μm. B) WGA staining indicating fungal hyphae colonization in the same set of genotypes at 5 dpi with *P. polysora* infection. Scale bars=100 μm.
